# Next-Generation Edible Packaging: Development of Water-Soluble, Oil-Resistant, and Antioxidant-Loaded Pouches for Use in Noodle Sauces

**DOI:** 10.3390/foods14061061

**Published:** 2025-03-20

**Authors:** Bahar Demircan, David Julian McClements, Yakup Sedat Velioglu

**Affiliations:** 1 Department of Food Engineering, Ankara University, Golbasi 06830, Türkiye; bdemircan@ankara.edu.tr; 2 Department of Food Science, University of Massachusetts, Amherst, MA 01003, USA

**Keywords:** biodegradable packaging, active packaging, curcumin, water-soluble pouch, lipid oxidation, natural antioxidants, controlled release

## Abstract

The development of sustainable biodegradable packaging materials is essential for enhancing food quality and shelf life while reducing plastic waste. This study explored polymer-based monolayer, composite, and bilayer films to produce water-soluble, oil-proof pouches. Single-serving seasoning oil pouches were prepared from bilayer films with polyvinyl alcohol (PVA) as the inner and sodium alginate (SA) as the outer layer. The PVA/SA films exhibited excellent UV protection, low oil permeability (0.18 × 10^−6^ g·mm/mm^2^·day), hydrophilic surface (water contact angle < 90°), and rapid solubility in hot water (87 ± 2 °C). Incorporating curcumin, a natural antioxidant, into PVA/SA films (Cur-PVA/SA) improved thermal stability, reduced light transmittance, and decreased water vapor permeability (0.28 × 10^−10^ g/m·Pa·s). Curcumin release followed a biphasic diffusion model, with 94.8% released at 96 h (diffusion coefficient: 1.30 × 10^−11^ m^2^/s), ensuring prolonged antioxidant activity. The Cur-PVA/SA pouches delayed lipid oxidation more effectively, with peroxide values of 6.48 and 10.35 meq/kg after 45 days at 35 °C, respectively. The Q_10_ model, which is commonly used to predict the shelf life of oils based on temperature-dependent oxidation rates, estimated that the oil packaged in Cur-PVA/SA pouches would remain stable for 12 months at 23 °C. This represents a 37% longer shelf life compared to oil packaged in PVA/SA pouches without curcumin. Cur-PVA/SA pouches also reduced noodle moisture migration, limiting weight loss to 2.73% over 14 days compared to 5.80% in controls. These findings highlight their potential as eco-friendly active packaging solutions.

## 1. Introduction

There is growing interest in developing biodegradable packaging materials to replace single-use plastics within the food industry so as to reduce their adverse environmental and sustainability impacts [1]. Indeed, it has been estimated that single-use plastic packaging accounts for around 40% of total global plastic production (390 million tons) [2]. A successful biodegradable packaging material should exhibit the mechanical, barrier, stability, and other functional characteristics required for commercial applications, which is often challenging [3]. For instance, achieving sufficient mechanical strength and flexibility in biodegradable films can be difficult, as many natural polymers lack the durability of synthetic plastics [4]. Additionally, creating effective Chemical Characteristics against moisture, oxygen, and oils is complex, as biodegradable materials often have higher permeability compared to conventional plastics. Stability is another critical factor, as biodegradable materials may degrade prematurely under certain environmental conditions, such as high humidity or temperature, compromising the shelf life of the packaged product [3]. Furthermore, balancing these properties while ensuring the material remains cost-effective and scalable for industrial production adds another layer of complexity. These challenges highlight the need for innovative approaches, such as combining different polymers or incorporating active compounds like preservatives, UV-blockers, plasticizers, and strengtheners, to develop biodegradable packaging that meets the rigorous demands of commercial applications [5,6].

In this study, we focused on the development of edible water-soluble pouches that could be used to deliver oils in food applications, with the aim of replacing the single-use plastic packages normally used for this purpose. For instance, these pouches could be used to deliver oil-based sauces to instant noodles when boiling water is poured on them. The oil-filled pouches and noodles would be packaged together for convenient preparation and consumption. Traditionally, these kinds of oil-based sauces are packaged within small non-edible pouches manufactured from synthetic plastics, such as polyethylene and polypropylene. These pouches are usually discarded after the oil-based sauce has been mixed with the food product. While cost-effective, these plastics present significant environmental and health concerns. Plastics contribute to environmental pollution during their production and disposal, they are difficult to recycle when contaminated with food residues, and they may release harmful substances into oils during prolonged contact [7,8,9]. Moreover, the packaged oils are highly susceptible to lipid oxidation during storage, which leads to rancidity and the formation of potentially toxic reaction products, thereby compromising food quality and consumer safety [10]. Conventional plastic packaging materials must also be opened by consumers, which leads to some inconvenience. Moreover, oil residues are often retained within the sachets, leading to considerable product waste [11]. These limitations highlight the need for the development of eco-friendly packaging solutions that can improve food quality, safety, and convenience. This kind of packaging material must exhibit several important quality attributes, including being comprised entirely of edible components, being capable of being hermetically sealed, maintaining its physical integrity during storage, protecting the oil from leakage and oxidation during storage, and rapidly dissolving once placed in hot water [12,13]. Moreover, the production of the packaging materials should be economical and scalable, so as to make the technology commercially viable.

Several researchers have already explored the potential of different kinds of edible pouches for oil packaging applications. For example, turmeric-enriched soybean polysaccharide/gelatin pouches have been shown to effectively inhibit lipid oxidation in soybean oil [8], whereas multilayered films assembled from pumpkin seed cake and corn zein have been shown to improve the oxidative stability of linseed oil [10]. Similarly, alginate-based pouches have been successfully used to preserve coconut oil during storage [7]. These studies highlight the potential of edible films as active packaging systems that deliver antioxidants, extend product shelf life, and reduce environmental impact [1]. However, most previous studies focus on film formulation and characterization without testing real-world applications. In contrast, this study utilizes a unique combination of water-soluble and oil-resistant polymers to optimize both dissolution and barrier properties. Polyvinyl alcohol (PVA), sodium alginate (SA), corn starch (CS), and hydroxypropyl methylcellulose (HPMC) were selected as the primary materials for the development of the edible pouches due to their complementary properties. CS and SA were chosen for their high water solubility, which is essential for creating pouches that dissolve rapidly in hot water. On the other hand, PVA and HPMC were selected for their excellent oil barrier properties, which are crucial for preventing oil leakage during storage. Individually, each polymer has certain limitations; for example, while CS and SA provide high water solubility, they may lack sufficient mechanical strength or oil resistance. Conversely, PVA and HPMC offer strong oil barrier properties but may not dissolve as quickly in water. By combining these materials into composite and bilayer films, we aimed to overcome the individual limitations of each polymer [14,15,16]. Additionally, the developed pouches were extensively characterized and tested with a real food product (noodle sauces), demonstrating their practical feasibility—an aspect often overlooked in prior research.

This study hypothesizes that biodegradable, water-soluble, and oil-proof layered pouches developed using PVA, SA, HPMC, and CS will exhibit superior barrier properties, enhanced oxidative stability, and rapid dissolution in hot water compared to conventional plastic-based packaging. Additionally, it is hypothesized that incorporating curcumin into the polymer matrix will enhance antioxidant activity, improve mechanical strength, and extend the shelf life of packaged oils.

## 2. Material and Methods

### 2.1. Materials

Polyvinyl alcohol (PVA, 341584, ρ: 1.19–1.31 g/m^3^, M_W_: 89–98 kDa), hydroxypropyl methylcellulose (HPMC, H9262, ρ: 80–120 cP, M_W_: ~26 kDa), sodium alginate (SA, W201502, ρ: 1 g/mL, M_W_: 12–40 kDA, M/G ratio: 1.56), corn starch (CS, S4126, (C_6_H_10_O_5_)_n_, ρ: 1.45–1.6 g/mL, containing approx. 73% amylopectin and 27% amylose), glycerol (GLY, G5516), and curcumin (CUR, Sigma-Aldrich C1386) were produced from Sigma-Aldrich (St. Louis, MO, USA). Sunflower oil-based seasoning oil was used for packaging in the produced pouches. Two types of precooked dried noodles (Indomie, Jakarta, Indonesia and Buldak, Atlantic Highlands, NJ, USA) that require addition of hot water prior to consumption were used for testing the efficacy of the biodegradable pouches. All chemicals used in the studies were of analytical purity. Double distilled water was used to prepare all solutions.

### 2.2. Preparation of Different Formulation Films

In this study, monolayer, composite, and bilayer films were prepared using a modified version of the methods described previously [17,18,19]:

- Monolayer Films: Solutions of 5% PVA and 2% CS were prepared by stirring in a heated water bath (95 °C) for 1 h. In addition, 2% HPMC and 2% SA solutions were prepared by stirring at room temperature overnight.
- Composite Films: Mixtures of 50:50 (*v*/*v*) of the following combinations were prepared: CS + PVA, CS + HPMC, SA + PVA, and SA + HPMC.
- Bilayer Films: The inner and outer layers were formed using a 50:50 (*v*/*v*) ratio of polymer solutions, producing the following combinations: PVA/CS, PVA/SA, HPMC/CS, and HPMC/SA. Bilayer films involved the outer layer being poured over the pre-dried inner layer and are referred to as “Inner/Outer”. Here, there is an expression from inside to outside, that is, the first mentioned layer is the inner surface.

All film solutions were homogenized at high speed for 3 min, and 30% (*w*/*w*, polymer basis) glycerol was added as a plasticizer. The solutions were then degassed to remove air bubbles before casting.

Different volumes (10, 15, or 20 mL) of film-forming solutions were poured into Petri dishes (100 × 15 mm, VWR International, Vienna). For bilayer films, the inner layer was first cast and then dried until reaching the gelation point, followed by the outer layer being poured on top and allowed to dry completely at room temperature. All dried films were conditioned at 25 ± 2 °C and 50 ± 2% relative humidity for 2 days before testing.

### 2.3. Determination of Optimum Film Casting Volume

The casting volume of the film-forming solution plays a significant role in the film’s properties, especially when producing pouches for packaging. To determine the ideal casting volume, the physical properties of 12 different formulations were tested after casting in three different volumes. The following properties were evaluated through visual observation: peeling behavior from Petri dishes, transparency, flexibility, durability, and surface appearance.

In addition, the thickness of each film was measured at 10 randomly selected points using a digital micrometer (0–25 mm, Schut Geometrical, Trossingen, Germany). The films were also weighed on a precision balance (SI-234, Denver Instrument, Denver, CO, USA) to further evaluate their physical characteristics.

### 2.4. Characterization of Films Prepared at Ideal Casting Volume

Based on the results of Section 2.3, the ideal film solution pouring volume was determined, and the following analyses were applied to the films prepared using this volume.

#### 2.4.1. Color

The color of each film was evaluated by measuring the CIELAB color parameters (L*, a*, b*) using a colorimeter (ColorFlex EZ, HunterLab, Reston, VA, USA). The total color difference (ΔE), yellowness index (YI), and whiteness index (WI) were calculated using Equations (1)–(3), respectively [20].

(1) $$∆E=\sqrt{{\left({∆L}^{*}\right)}^{2}+{\left({∆a}^{*}\right)}^{2}+{\left({∆b}^{*}\right)}^{2}}$$

(2) $$YI=\frac{{142.86b}^{*}}{L^{*}}$$

(3) $$WI=100-\sqrt{{\left({100-L}^{*}\right)}^{2}+{\left(a^{*}\right)}^{2}+{\left(b^{*}\right)}^{2}}$$

#### 2.4.2. Barrier Properties

To evaluate the light barrier properties, the film samples were cut to dimensions of 1 × 4 cm and then placed in a quartz cuvette. The film’s ability to block UV and visible light was assessed by measuring transmittance (%) across the wavelength range from 200 to 800 nm using a UV-visible spectrophotometer (Genesys 150, Thermo Scientific, Madison, WI, USA). The UV shielding capacity was taken to be the light transmittance measured at 280 nm [20]. Additionally, the transparency of the film was calculated using the transparency value (A/mm), which is the ratio of the absorbance measured at 600 nm (A) to the measured film thickness (mm). A higher transparency value indicates a lower film transparency [21].

The oil permeability (OIP) was determined by placing the film sample onto pre-equilibrated and pre-weighed filter paper (P-8 coarse grade, Fisher Scientific, Cleveland, OH, USA), then adding 3 mL of sunflower oil on top. The setup was left for 7 days at 23 ± 2 °C and 50 ± 5% relative humidity. After the incubation period, the filter paper was reweighed, and the OIP (g·mm/mm^2^·day) was calculated using Equation (4), as reported by Rosenbloom and Zhao [11].

(4) $$Oil\;permeability=\frac{\left[mass\;change\left(g\right)\times film\;thickness\left(mm\right)\right]}{\left[film\;area\left({mm}^{2}\right)\times time\left(days\right)\right]}$$

The water vapor permeability (WVP) of the film was determined using the wet cup method. A 10 mL test tube with a diameter of 14 mm was filled with 6 mL of distilled water. The test film was then placed over the mouth of the test tube, securely fastened with parafilm along the edges, and then weighed. The tube was then placed in a desiccator containing silica gel to maintain a relative humidity of 0% at 30 ± 0.5 °C. The weight of the tube was recorded at 12 h intervals for 3 days. The WVP (g/m·Pa·s) was calculated using the formula provided by Wu et al. [22].

#### 2.4.3. Chemical Characteristics

Fourier transform infrared (FTIR) spectra of films were acquired using an infrared spectrophotometer (IR Prestige 21^®^, Shimadzu, Kyoto, Japan) equipped with an ATR accessory. A scan rate of 32 scans/sample was used over the wavenumber range from 600 to 4000 cm^−1^ at a resolution of 4 cm^−1^ [8].

#### 2.4.4. Thermal Properties

The thermal stability and decomposition of the films were determined using a Thermogravimetric Analysis (TGA) and Differential Scanning Calorimetry (DSC) (SDT650 and DSC25, TA Instruments, Waltham, MA, USA). For TGA, approximately 10 mg of each film sample was placed in a measurement crucible and then its mass was recorded when it was heated from 30 to 600 °C at a rate of 10 °C/min. The resulting mass–temperature profile was used to analyze the thermal stability and decomposition of the film. For DSC, approximately 10 mg of film sample was accurately weighed into an aluminum pan and hermetically sealed. Each sample was then heated from 20 to 320 °C at a rate of 10 °C/min, and the enthalpy change was recorded as a function of temperature. An empty aluminum pan was used as a reference. Nitrogen gas was flushed over the samples at flow rates of 20 and 100 mL/min for TGA and DSC, respectively, to maintain a controlled atmosphere and prevent thermo-oxidative reactions [12,20].

#### 2.4.5. Morphological Characteristics

The morphology of the films was characterized by acquiring scanning electron microscopy (SEM) images using a scanning electron microscope (JCM-6000 NeoScope, JEOL, Tokyo, Japan). The samples were coated with about 10 nm of gold at 40 mA/mbar (Cressington 108Auto, Redding, CA, USA) for 60 s, and cross-section and surface images were recorded under high vacuum mode at 10 kV at different magnifications [20].

#### 2.4.6. Water Contact Angle Measurement

The surface wettability of the films was characterized by measuring their water contact angle (WCA). Initially, small aliquots (15 μL) of distilled water were dropped from 10 mm above the film surface at 5 random points using an automated microsyringe (diameter of 0.9 mm). The WCA of each water drop was then measured using a drop shape analyzer (DSA30R, Krüss, Hamburg, Germany). Images of the water droplets were recorded as soon as they came into contact with the film, as well as 5 and 15 s later. The WCA was determined from the digital images obtained using the instrument software (Advance Software V1.7, Hamburg, Germany), as described previously [8].

#### 2.4.7. Water Solubility

Films were cut into 3 × 3 cm squares and then placed in a beaker containing 30 mL of water at either 25 ± 2 °C or 87 ± 2 °C and then stirred with a magnetic stirrer at 200 rpm. The time taken for the films to completely dissolve (the point at which they became invisible) was recorded using a stopwatch. In films with a dissolution time longer than 60 s, the analysis was stopped at 60 s, and their solubility within 60 s was determined. The solubility (%) values were calculated by filtering the beaker’s content through a coarse filter paper and then drying the residual film piece in an oven (20GC, Quincy Lab, Burr Ridge, IL, USA) until it reached constant weight [8].

### 2.5. Preparation and Characterization of Antioxidant-Enriched Films

The optimum film formulation used to produce the biodegradable pouches was established based on the previous analyses. A natural antioxidant (curcumin) was included in this formulation to obtain the antioxidant-enriched films. For this purpose, after the glycerol addition step in the film-forming method described earlier, curcumin was added at 0.5% (*w*/*v*) final concentration by previously dissolving it in ethanol. Then, the solutions were homogenized, degassed, poured into Petri dishes at optimal casting volume, and dried at room temperature [23].

The enriched films were then characterized using the same analyses described above (Section 2.3 and Section 2.4). In addition, the following analyses were applied to each antioxidant-loaded film.

#### 2.5.1. Curcumin Diffusion Analysis

A pre-weighed film (app. 0.3 g mg) was placed in 50 mL of ethanol: water (1:1, *v*/*v*) and then stirred using a magnetic stirrer at room temperature. Samples (3 mL) were periodically withdrawn and assayed for curcumin release until steady state conditions were reached. The amounts of curcumin released at different time intervals were determined by measuring the absorbance at 279 nm using a UV-visible spectrophotometer. The released amount of curcumin (%) was determined using a curcumin standard curve (Abs = 0.906[Cur] + 1.1546, R^2^ = 0.9164).

The effective diffusion coefficient of the antioxidants in the film was determined from the release kinetics assuming a Fickian release mechanism under transient state conditions:

$$\frac{C_{t}}{C_{p}}=\frac{4}{L_{p}}\times {\left(\frac{D_{t}}{\pi }\right)}^{0.5}\;for\;0\leq \frac{C_{t}}{C_{p}}\leq 0.6$$

Here, *C_t_* is the concentration of curcumin in the dissolution medium at time *t* (s), *C_P_* is the maximum concentration in the dissolution medium when equilibrium is reached, *L_p_* is film thickness, and *D* is the diffusion coefficient (m^2^/s). The value of *D* was estimated from the slope of a plot of *C_t_*/*C_p_* versus *t*^0.5^, as described previously [24].

#### 2.5.2. Release Study to Different Food Simulants

The release study to different food simulants was conducted based on a modified method adapted from Liu et al. [25] and Gonçalves et al. [26]. The three food simulants—distilled water (DW), 95% ethanol (95E), and 50% ethanol (50E)—were selected to represent aqueous, fatty, and emulsified food systems, respectively. These simulants are widely used in migration studies of edible films to evaluate the release of active compounds under conditions that mimic real food environments. Briefly, film pieces (3 × 3 cm) were immersed in 30 mL of food simulants placed in 50 mL glass vials with screw caps. Release tests were conducted in the dark at 25 °C with constant stirring at 50 rpm. The food simulant was collected, and its total phenolic content (TPC) and antioxidant activity (AOA) were determined at predetermined time intervals for 7 days. All analyses were measured in triplicate with three different vials.

#### 2.5.3. Total Phenolic Content and Antioxidant Activity

The TPC released from the film was determined by the Folin–Ciocalteu method [25]. Each food simulant (20 μL) was thoroughly mixed with 100 μL of Folin–Ciocalteu reagent (10% *v*/*v*). Then, 80 μL of 7.5% (*w*/*v*) sodium carbonate was added to the mixture, and the sample was allowed to stand for 60 min at room temperature. The absorbance was then measured at 725 nm using a UV-visible spectrophotometer. The total phenol content (mg gallic acid eq. (GAE)/g film) was calculated using a gallic acid standard curve (Abs = 0.008[GA] + 0.0118, R^2^ = 0.9997).

The AOA released from the film matrix was evaluated using the 2,2-diphenyl-1-picrylhydrazyl (DPPH) assay [25]. Food simulant (80 μL) was mixed with 100 μL (0.1 mM) of DPPH dissolved in methanol. The mixture was then kept in the dark for 30 min at room temperature. The absorbance was measured at 517 nm using a UV-visible spectrophotometer, and the DPPH radical scavenging activity (%) was determined.

### 2.6. Production of Film Pouches and Their Usage for Single-Serving Seasoning-Oil Packaging

Control and enriched films were heat-sealed for 2 s with an impulse sealer (4.7″ heating strip, Tanyaxue brand) on three sides to form an open rectangular (35 × 50 mm) pouch, taking into account the inner and outer surfaces of the films. Seasoning oil was then poured into the pouch on the open side, and the fourth side was sealed to form a closed pouch.

Pouches loaded with seasoning oil were placed in a beaker containing 300 mL of hot water at 87 ± 2 °C, under constant 200 rpm stirring. The complete dissolution of the pouches and the dispersion of all the oil mixture into the water were recorded with a digital camera. The dissolution time was recorded using a stopwatch, as described previously [8,27]. The performance of these pouches was also tested in a similar manner during the noodle preparation phase.

### 2.7. Storage of the Seasoning Oil-Containing Pouches in Contact with the Noodles

The produced oil-loaded pouches (control and antioxidant-enriched ones) were placed on the noodles and then stored at room temperature to simulate their application in commercial products. During storage, the weight change in the oil-containing pouches and the water activity of the noodles were analyzed. The aim of these experiments was to determine whether the noodles would absorb any moisture from the pouches and whether the pouches could prevent moisture migration.

### 2.8. Quality Changes in Oil Packaged in Pouches During Storage

For these experiments, sunflower oil was added to the pouches (control and antioxidant-enriched ones) instead of seasoning oil to eliminate the possible antioxidant effects of the spices on the oxidation study. The oil-containing pouches were stored in the dark at a relative humidity of 50 ± 2%, which was similar to the internal RH of instant noodle packages, at 35 ± 2 °C for 45 days to accelerate oxidation. The quality attributes of the oils in the pouches were measured throughout storage, including the peroxide value (PV) and thiobarbituric acid reactive substances (TBARS) value, using the methods described previously [28]. The equivalent degree of lipid oxidation that would have occurred if the samples were stored under ambient conditions was estimated using the *Q*_10_-quotient model: *Q*_10_ = (*k*_2_/*k*_1_)^10/(*T*2−*T*1)^ [11]. Using the *Q*_10_ constant experimentally determined from the peroxide values (*k*_1_) measured at the test temperature (T_1_, 35 °C), the peroxide values estimated (*k*_2_) at ambient temperature (T_2_, 23 °C) were calculated.

### 2.9. Statistical Analysis

All experiments were performed with 3 parallels and 3 replications per sample. The mean and standard deviation was then calculated from these measurements. The collected data underwent factorial analysis of variance (ANOVA) followed by Duncan’s test for comparison. All statistical analyses were conducted using SPSS 22.0 software (SPSS Inc., Chicago, IL, USA), with a significant difference assumed to occur when *p* < 0.05.

## 3. Results and Discussion

### 3.1. Determination of Optimum Film Casting Volume

Initially, the impact of the casting volume and formulation on the physical characteristics of the films was assessed. The casting volume plays a critical role in determining the mechanical properties, thickness, and flexibility of the films, which are essential for designing pouches suitable for food packaging [29,30]. Films prepared from 10 mL of polymer solution were too thin, weak, and inhomogeneous, making them unsuitable for practical applications due to their poor durability and ease of tearing. Conversely, films prepared from 20 mL of polymer solution were too thick, exhibited a hazy appearance, and had reduced transparency and higher residual moisture, especially when used in composite and bilayer formulations. These thick films were also less flexible and more difficult to peel from the Petri dishes, which could complicate the pouch production process [31].

The films prepared from 15 mL demonstrated the optimal balance of properties, including good durability, transparency, and ease of peeling. For example, SA films cast at 15 mL were transparent, easily peelable, and durable, while PVA films displayed transparency, flexibility, and a plastic-like appearance. Similarly, composite films such as CS+HPMC and SA+PVA cast at 15 mL had good durability, minimal haze, and appropriate thickness. Bilayer films like HPMC/CS and HPMC/SA prepared using 15 mL of the polymer solutions exhibited uniform drying and adequate thickness despite some minor surface imperfections.

Thickness and weight measurements also indicated that 15 mL was the most suitable casting volume for film preparation (Table 1). These films demonstrated consistent thickness and moderate weight, compared to the thinner and lighter films from 10 mL or the excessively thick and heavy films from 20 mL. The selection of 15 mL as the optimal casting volume was based on the need for films that are neither too thin (to avoid tearing) nor too thick (to maintain flexibility and transparency), while also ensuring easy peeling from the Petri dishes. This balance of properties is crucial for the production of pouches that can effectively protect and deliver seasoning oils [1].

### 3.2. Determination of Optimum Physicochemical Characteristics of Films

**Figure 1:**
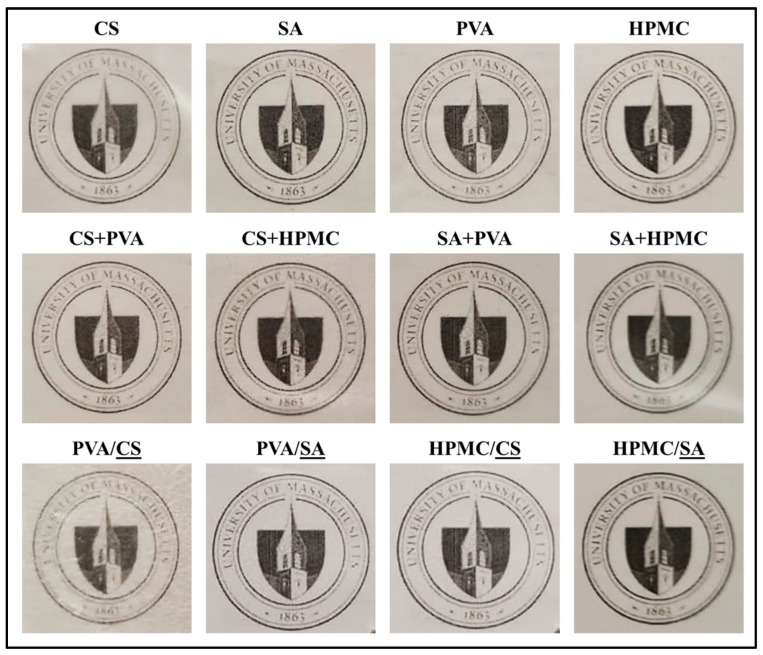
Appearance of 15 mL volume of cast films on logo printed paper (CS: corn starch, SA: sodium alginate, PVA: polyvinyl alcohol, HPMC: hydroxypropyl methylcellulose, “+”: composite films (mixture), “/”: bilayer films (inner/outer)). The underlined layer represents the photographed surface.

This series of experiments was performed to identify the biodegradable films that had the optimum physicochemical characteristics for application as hot water-soluble pouches for protecting and delivering seasoning oils. Consequently, the impact of film composition and structure (monolayer, composite, or bilayer) on their physicochemical properties was examined, including their optical, barrier, chemical, thermal, structural, surface, and solubility properties. These experiments were all carried out using films prepared with a casting volume of 15 mL (Figure 1). Figure 1 illustrates the appearance of the films cast on logo-printed paper, demonstrating how the films affect the visibility of the underlying design. For instance, the PVA/CS film shows a blurred and mosaic-like appearance, indicating lower transparency, while the PVA/SA film allows the logo and text to be clearly visible, demonstrating higher transparency. Similarly, the PVA film has a lighter color, making the background more visible, whereas the CS+HPMC film has a darker color, reducing the visibility of the background. These visual differences highlight the variations in film transparency and their potential impact on applications where visual clarity is important, such as food packaging.

#### 3.2.1. Color

The color parameters (L*, a*, b*, ΔE*, YI, WI) highlighted significant variations among film formulations (Table 2), reflecting the influence of polymer composition. The CS films exhibited moderate lightness (L* = 31.96) with near-neutral chromaticity (a* = 0.39; b* = −0.33), indicating slight translucency. The SA films displayed the lowest lightness (L* = 4.75) and most negative YI (−15.84), making them the darkest and most yellowish films.

The PVA and HPMC films had higher lightness values (L* = 11.54 and L* = 37.09, respectively), with HPMC being the most transparent (WI = 37.08). Composite films like CS+PVA and CS+HPMC exhibited intermediate lightness values (L* = 14.85 and L* = 15.83), balancing their components’ optical properties. The bilayer films, such as PVA/CS and PVA/SA, also displayed intermediate lightness values, with the PVA/SA films appearing brighter (L* = 34.58). These results align with previous studies that have also reported that the polymer composition of biodegradable films influences their optical characteristics [32,33,34].

#### 3.2.2. Barrier Properties

**Figure 2:**
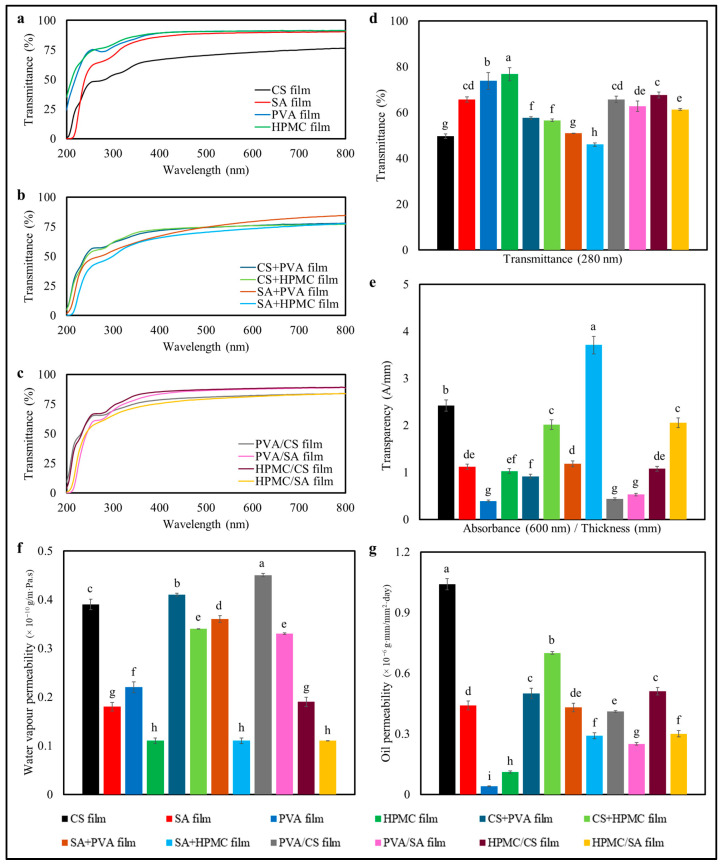
Light transmittance (**a**–**e**), water (**f**) and oil permeability (**g**) of films (CS: corn starch, SA: sodium alginate, PVA: polyvinyl alcohol, HPMC: hydroxypropyl methylcellulose, “+”: composite films (mixture), “/”: bilayer films (inner/outer)). Different letters indicate significant variances between samples according to the Duncan test (*p* < 0.05).

The composition of the biodegradable films also impacted their light transmittance profiles over the wavelength range from 200 to 800 nm (Figure 2a–c), which would be expected to impact the photostability of any substances prone to degradation when exposed to UV or visible light. All the films strongly absorbed UV light (200–280 nm), with the transmittance values being below 10%, confirming their effective UV-blocking capacity. However, the transmittance of visible light (400–800 nm) varied considerably among the different formulations. The HPMC and PVA films had the highest transmittance (>60%), while the SA-based films and the composite films (e.g., CS+HPMC) showed moderate transmittance (30–50%) (Figure 2d). The bilayer films, like PVA/CS and PVA/SA provided the most effective light barriers, with transmittance values below 30% across the entire visible spectrum.

Films with higher transparency, such as HPMC (0.40 A/mm) and PVA (0.55 A/mm), would be expected to display weaker light-shielding effects (Figure 2e). In contrast, the SA-based films, including the bilayer films (such as PVA/SA), exhibited the lowest transparency values (<0.20 A/mm), confirming their effectiveness in reducing light transmission. Our results are consistent with those reported by previous researchers on biodegradable films [35,36,37,38].

In terms of the WVP (Figure 2f), the PVA-based and CS+PVA composite films exhibited the highest resistance to water transfer (lowest WVP), while the SA-based films exhibited the lowest (highest WVP) (Figure 2). The HPMC- and CS-based films exhibited intermediate WVP values, suggesting their suitability for moderate moisture-sensitive applications. Kader et al. [15] reported that the water resistance in composite and bilayer films increased with increasing crosslinking of the polymers, whereas Khater et al. (2023) reported decreasing WVP values in composite films with increasing thickness.

The OIP tests (Figure 2g) revealed that the HPMC- and SA-based films, including their composite and bilayer forms, provided the highest resistance to oil penetration, while the CS-based films showed moderate resistance. Similarly, Xu et al. [39] reported that composite films were the most suitable for oily foods. PVA-only films exhibited the lowest oil resistance, which would limit their use in fatty food packaging applications.

These findings emphasize the impact of polymer composition on barrier properties, enabling film customization for specific applications such as light shielding, water vapor resistance, and oil barrier performance. Similar trends have been observed in previous studies [39,40].

#### 3.2.3. Chemical Characteristics

**Figure 3:**
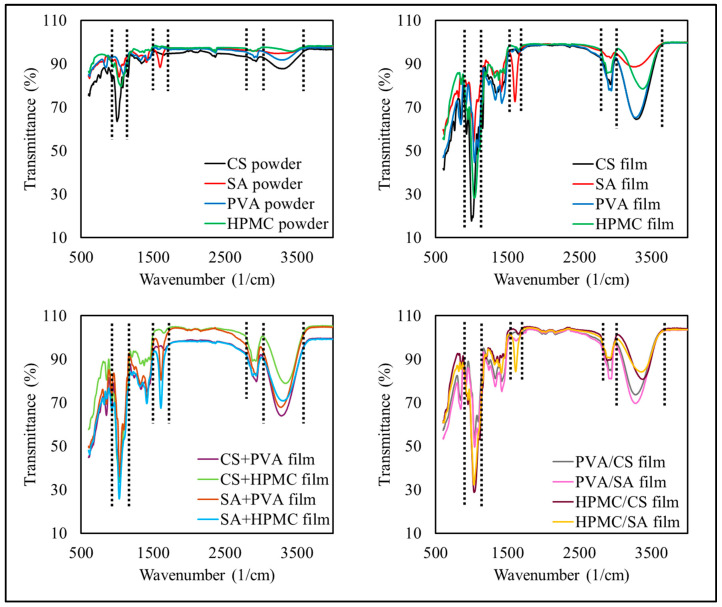
FTIR spectra of films (CS: corn starch, SA: sodium alginate, PVA: polyvinyl alcohol, HPMC: hydroxypropyl methylcellulose, “+”: composite films (mixture), “/”: bilayer films (inner/outer)).

FTIR spectroscopy was used to provide insights into the nature and interactions of the functional groups within the polymer matrices of the different films (Figure 3). The position and intensity of the peaks within the spectra depend on the types and amounts of polymers present, as well as their interactions with each other.

For the CS-based films, broad peaks were observed at 3280–3400 cm^−1^, which correspond to hydrogen-bonded hydroxyl groups [15]. The characteristic peak observed at 1650 cm^−1^ was attributed to bound water, while the peaks observed at 1580 and 1310 cm^−1^ were associated with the stretching vibrations of the C–O bonds in the C–O–C and C–O–H groups, which is consistent with the findings of Ren et al. [41].

For the SA-based films, strong absorption bands were observed at 1600 and 1410 cm^−1^, which were attributed to the asymmetric and symmetric stretching vibrations of carboxylate groups (COO^−^) and are characteristic of ionic binding [42]. The broad peak observed at 3000–3500 cm^−1^ was attributed to O–H stretching, which are associated with the hydroxyl groups in the alginate structure.

For the PVA-based films, the characteristic peaks observed at 3300–3400 cm^−1^ were attributed to O–H stretching, those at 2940 cm^−1^ were attributed to C–H stretching, and those at 1140 cm^−1^ were attributed to C–O stretching. The smaller peak observed at 1730 cm^−1^ was attributed to the presence of acetate groups, reflecting the semi-crystalline structure of PVA, as noted by Kader et al. [15].

For the HPMC-based films, a strong O–H stretching peak was observed at 3300 cm^−1^ and ether bond peaks were observed at 1050–1150 cm^−1^ (C–O–C stretching), which are consistent with the main functional groups found in polysaccharides [43].

In the composite films (e.g., CS+PVA, CS+HPMC, and SA+PVA) and bilayer films (e.g., PVA/CS, PVA/SA, and HPMC/CS), peaks originating from the separate components were observed in the spectra, indicating that both polymers had been successfully incorporated into the films. For instance, in CS+PVA films, the O–H and N–H stretching bands from CS were observed alongside the C–H and C–O stretching peaks of PVA, indicating good compatibility between the two polymers [15]. Similarly, in SA+PVA films, peaks at 1600 cm^−1^ (COO^−^ asymmetric stretch) and 1140 cm^−1^ (C–O stretch) confirmed the presence of both polymers. Moreover, no new peaks were observed in the spectra, which indicates that there were no strong chemical reactions between the polymers.

The bilayer films, such as PVA/CS and HPMC/SA, retained the distinct functional groups of each layer without forming new peaks indicative of chemical bonding, confirming that physical rather than chemical interactions governed their structure.

Overall, the FTIR spectra demonstrated that the characteristic polymer peaks were preserved in the composite and bilayer films, which suggests that the dominant interactions between the components were physical (rather than chemical), highlighting the compatibility of the polymers used in these formulations.

#### 3.2.4. Thermal Properties

**Figure 4:**
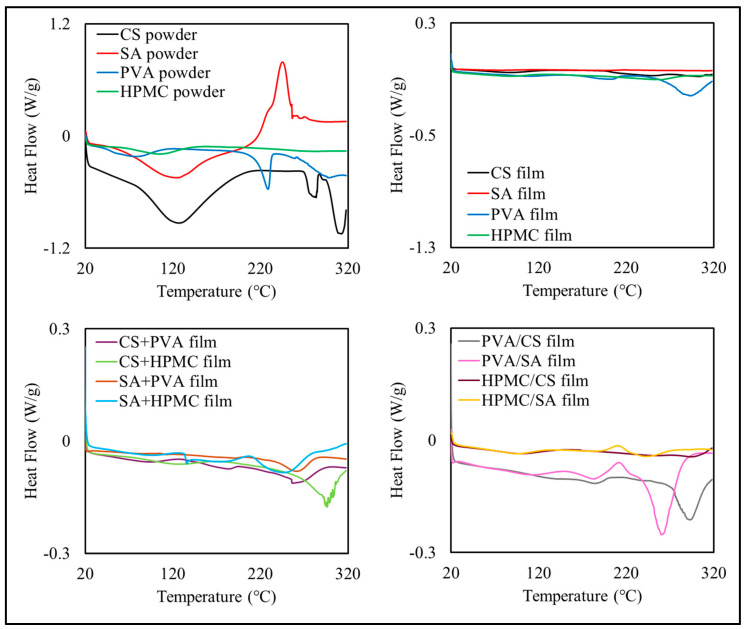
DSC graphs of films (CS: corn starch, SA: sodium alginate, PVA: polyvinyl alcohol, HPMC: hydroxypropyl methylcellulose, “+”: composite films (mixture), “/”: bilayer films (inner/outer)).

The DSC thermograms of the original polymer powders and the films assembled from them suggested that several thermal events occurred during heating (Figure 4), which may have been a result of glass-rubbery transitions, polymer melting, polymer–polymer interactions, evaporation, and thermal degradation reactions. Typically, moisture would be expected to evaporate around and above 100 °C, which would lead to an endothermic peak. The melting of crystalline regions would be expected to lead to an endothermic peak, while the thermal degradation of polymers and other substances would be expected to give an exothermic peak.

There were clearly appreciable differences between films with different compositions, which indicate that the individual polymers behaved differently, as well as their combinations with each other. In general, however, it was difficult to assign the origin of the observed thermal events to precise molecular phenomena in the different films.

**Figure 5:**
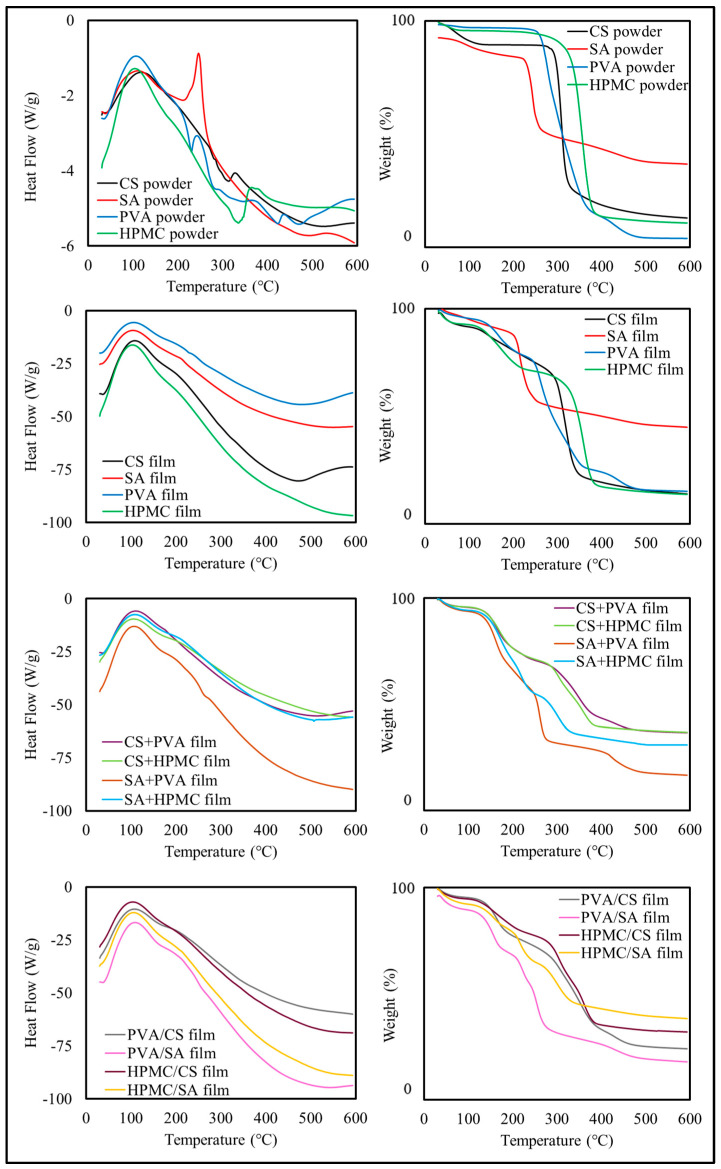
TGA graphs of films (CS: corn starch, SA: sodium alginate, PVA: polyvinyl alcohol, HPMC: hydroxypropyl methylcellulose, “+”: composite films (mixture), “/”: bilayer films (inner/outer)).

The TGA thermograms also highlighted differences in the thermal stability and degradation patterns of the different films (Figure 5). In general, there were three major weight loss regimes observed in the films during heating, which is consistent with previous studies [44]. The first regime (70–150 °C) was observed in all formulations and was mainly attributed to the evaporation of residual water trapped within the films [45,46]. The second regime (250–350 °C) was mainly attributed to the thermal decomposition of the polymer backbones [47]. The third regime (400–700 °C) was mainly attributed to thermal degradation of the remaining organic matter.

The CS and SA films only exhibited a two-step degradation profile, with moisture loss followed by significant thermal degradation of their polysaccharide backbones. The PVA films showed a single sharp degradation step from 250 to 350 °C, indicating their nearly complete thermal decomposition. The HPMC films demonstrated moisture evaporation followed by significant thermal degradation between 250 and 370 °C.

The CS+PVA and SA+PVA films exhibited a three-step degradation pattern, combining the thermal characteristics of their components and displaying enhanced thermal stability, suggesting partial synergistic interactions. The bilayer films, such as PVA/CS and PVA/SA, exhibited a combination of the thermal degradation profiles of their two layers, confirming their additive thermal properties [45,48].

Overall, the composite and bilayer films demonstrated enhanced thermal stability and additive properties, making them more resilient at higher temperatures. These findings suggest their suitability for applications requiring thermal resistance, particularly in food packaging environments.

#### 3.2.5. Morphological Characteristics

**Figure 6:**
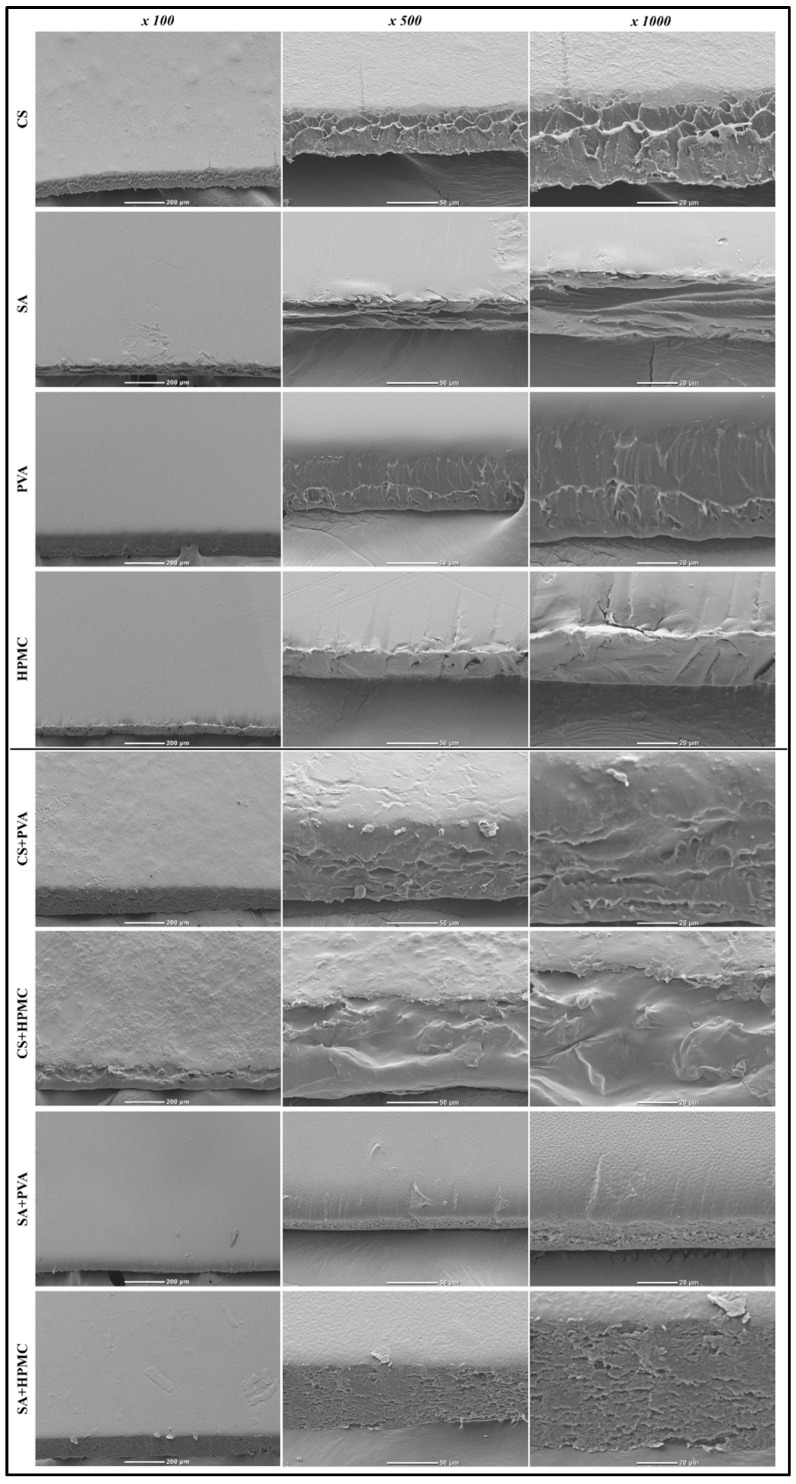
Surface and cross-sectional SEM images of monolayer and composite films (CS: corn starch, SA: sodium alginate, PVA: polyvinyl alcohol, HPMC: hydroxypropyl methylcellulose, “+”: composite films (mixture)).

**Figure 7:**
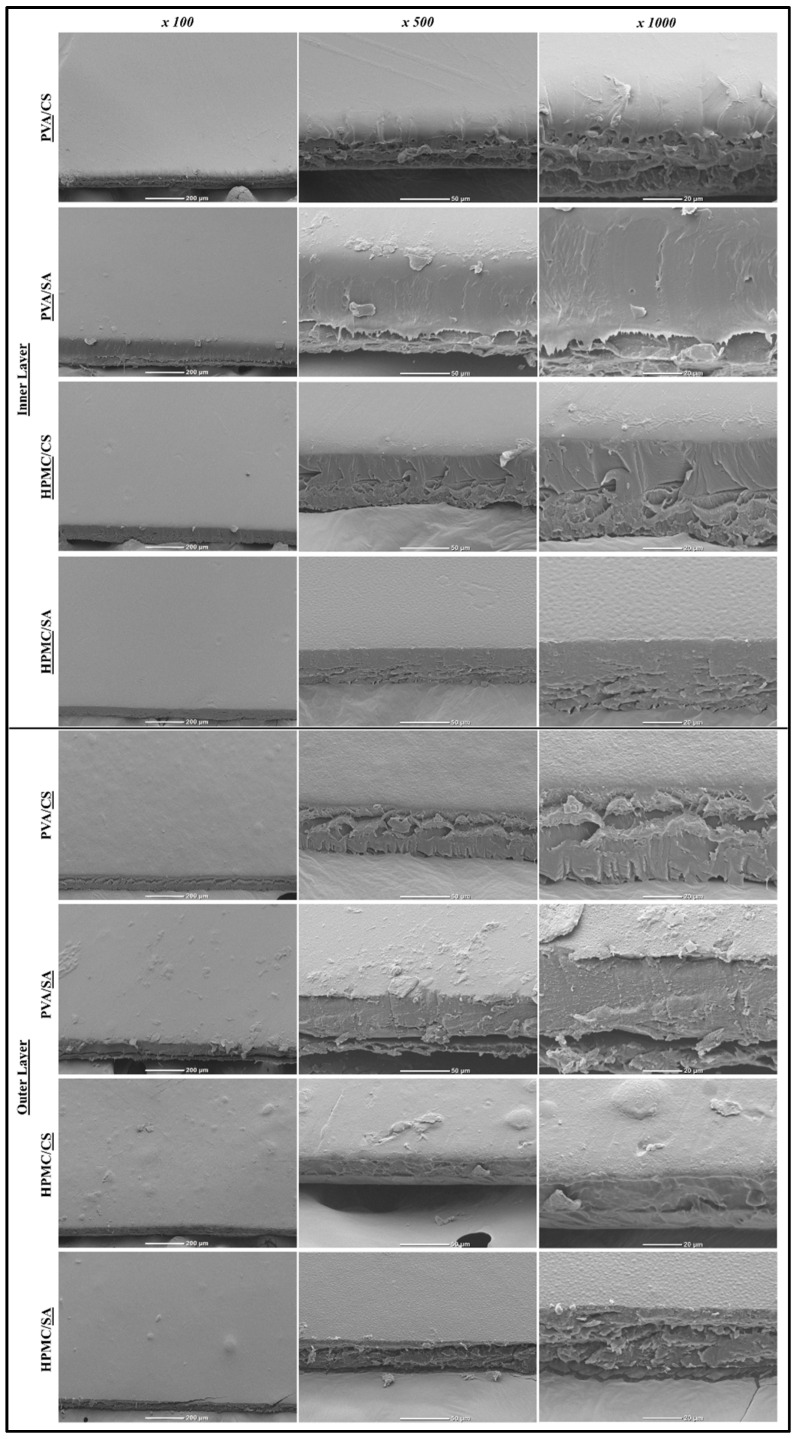
Surface and cross-sectional SEM images of bilayer films (CS: corn starch, SA: sodium alginate, PVA: polyvinyl alcohol, HPMC: hydroxypropyl methylcellulose, “/”: bilayer films (inner/outer)). The underlined layer represents the photographed surface.

The SEM images of the monolayer, composite (Figure 6), and bilayer films (Figure 7) showed that their surface and cross-sectional structure depended on their compositions.

All the monolayer films had relatively smooth and uniform outer surfaces, indicating that the polymers formed a uniform gel network [49]. Notably, the PVA films had glossy uniform surfaces, which is characteristic of their semi-crystalline nature. Some of the composite films also had relatively smooth surfaces, but others had relatively heterogeneous surfaces. For instance, the SA+PVA and SA+HPMC films had relatively smooth surfaces, which suggests that these polymer pairs were relatively miscible. In contrast, the CS+PVA and CS+HPMC films had relatively rough surfaces, which suggests these polymer pairs were not fully miscible.

At higher magnifications (500× and 1000×), the cross-sectional images of the films provided insights into their internal structures. The CS films had a compact and dense cross-sectional morphology, suggesting that a strong polymer network had been formed with good mechanical robustness [49]. The SA films had a more layered and porous cross-sectional morphology, suggesting a weaker structural cohesion. The PVA films had relatively compact and smooth morphologies, which is consistent with their semi-crystalline nature. The HPMC films had relatively wrinkled cross-sectional morphologies. The cross-sectional images of the composite films showed that some of them had considerably more heterogeneous structures than those of the monolayer films. For instance, the CS+PVA and CS+HPMC films had relatively heterogeneous structures, suggesting good some incompatibility between the two polymers used to assemble them. In addition, the SA+HPMC films exhibited some layering and fracturing, suggesting polymer incompatibility. In contrast, the SA+PVA films had a more uniform structure, suggesting that they had better polymer compatibility.

The SEM images of the bilayer films showed that their inner and outer layers had different structures, which depended on the properties of the individual films they were assembled from. For instance, PVA outer layers of the PVA/CS and PVA/SA films had relatively smooth and dense surfaces, which are characteristic of the monolayer films formed by PVA. Consequently, these outer layers would be expected to provide good barrier properties to the bilayer films. In contrast, the inner structures of the CS or SA inner layers of these films appeared rougher and more heterogeneous, which may contribute to their flexibility and porosity. The cross-sectional images of the bilayer films showed well-defined boundaries between the different layers, with minimal interfacial gaps, indicating strong adhesion between the different layers.

In summary, the SEM images show that the microstructure of the different films depended on their polymer composition and fabrication method (bilayer versus composite). As the microstructure of films impacts their physicochemical and functional attributes, it may, therefore, be possible to create biodegradable films with different properties by varying their composition and fabrication method.

#### 3.2.6. Water Contact Angle Measurements

**Figure 8:**
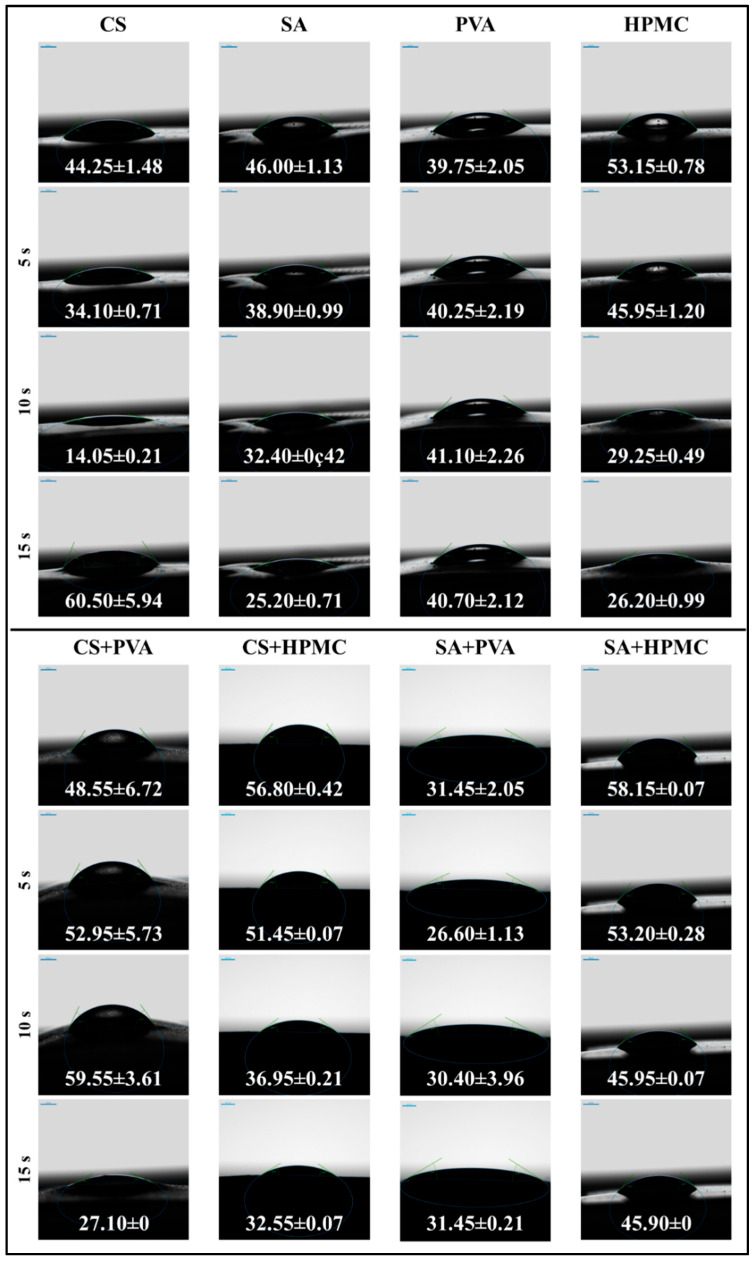
Water contact angle of monolayer and composite films (CS: corn starch, SA: sodium alginate, PVA: polyvinyl alcohol, HPMC: hydroxypropyl methylcellulose, “+”: composite films (mixture)).

**Figure 9:**
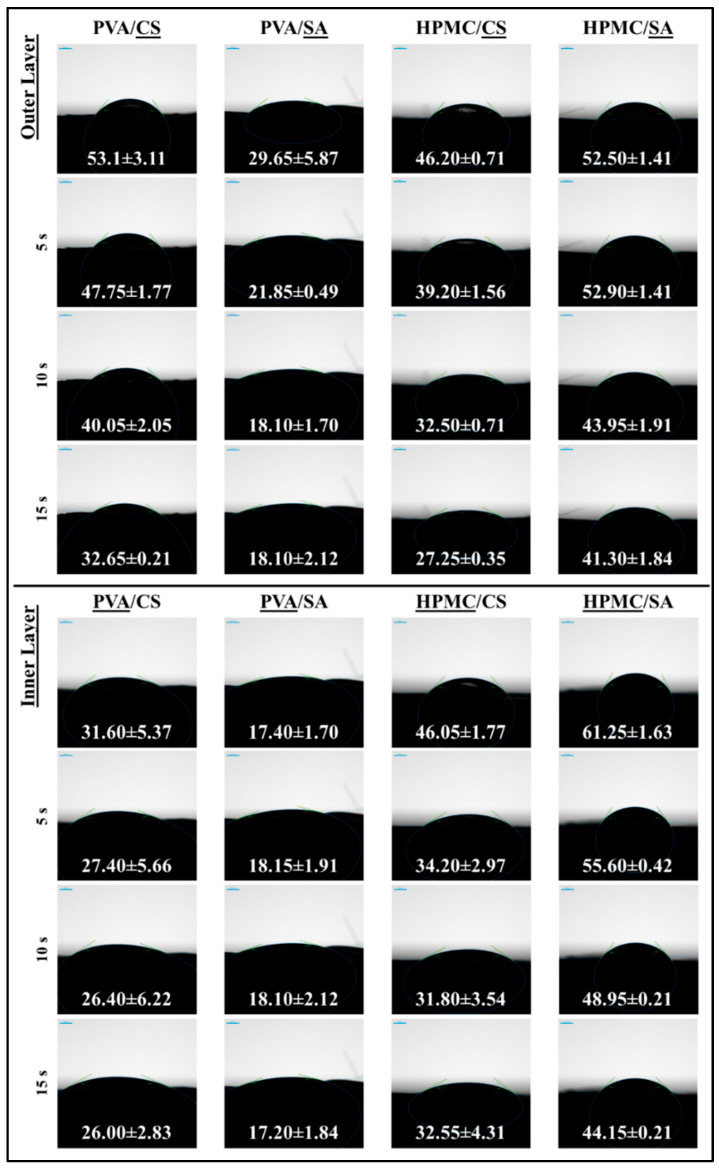
Water contact angle of bilayer films (CS: corn starch, SA: sodium alginate, PVA: polyvinyl alcohol, HPMC: hydroxypropyl methylcellulose, “/”: bilayer films (inner/outer)). The underlined layer represents the layer that the water drop contacts.

The wettability of the films was characterized by measuring the WCA when a drop of water was placed on top of them (Figure 8 and Figure 9). The WCA is a widely recognized measure of a film’s hydrophilicity or hydrophobicity, with values below 90° indicating predominantly hydrophilic surfaces and values above 90° indicating predominantly hydrophobic surfaces [50,51]. In these experiments, the initial WCA values were measured, as well as their change over time. The initial value is likely to depend on the initial wettability of the films, whereas changes over time are likely to reflect alterations in film properties due to penetration of water into the films, followed by dissolution of the polymers.

The wettability of the different monolayer films clearly depended on their composition (Figure 8). The CS films initially had a contact angle of 44.25°, but this rapidly decreased to around 34.10° at 5 s and 14.05° at 10 s, before rapidly rising to 60.50° at 15 s. These results can be attributed to rapid absorption of water by these films followed by surface instability [52].

The SA films demonstrated a consistent decline in WCA from 46.00° to 25.20° over time, reflecting alginate’s hydrophilic nature and gradual water penetration [53]. Conversely, the PVA films exhibited little change in WCA over time, with the value remaining around 39.75°, indicating limited water absorption, which can be attributed to the semi-crystalline structure of this polymer. The WCA of the HPMC films decreased from around 53.15° to 26.20°, indicating that they became more hydrophilic, which can also be attributed to the hydrophilic nature of this polymer and gradual water penetration [33].

The composite films exhibited wettability values that depended on those of their individual components (Figure 8). For instance, the CS+PVA films initially increased in contact angle from 48.55° to 59.55° at 10 s before declining sharply to 27.10° at 15 s, reflecting a slight initial decrease in hydrophilicity followed by a steep increase, which may have been due to strong water absorption after longer contact times. The CS+HPMC films showed a gradual decline from 56.80° to 32.55°, indicating enhanced water resistance compared to pure CS films. The contact angles of the SA+PVA films remained relatively constant over time (around 31.45°), suggesting that they exhibited little water adsorption over the measurement period [54]. The SA+HPMC films demonstrated a steady decline from 58.15° to 45.90°, suggesting that they became slightly more hydrophilic due to water adsorption.

In general, the wettability of bilayer films depends on which side of the film is directly in contact with water. For this reason, the WCA values of both sides of the bilayer films were measured (Figure 9). In the images of the water drops in contact with the bilayer films, the layer that is underlined is the one that is in direct contact with water. The CS outer layer of the PVA/CS films exhibited a steady decline in contact angle from 53.10° to 32.65°, which is indicative of the hydrophilic nature of the corn starch film and its ability to absorb water. The alginate outer layer of the PVA/SA films exhibited a steep decrease in contact angle from 29.65° to 18.10°, which can be attributed to its strong hydrophilic nature and ability to adsorb water [55].

The CS outer layer of the HPMC/CS films exhibited an appreciable decline in contact angle from 46.20° to 27.25°, which can again be attributed to the relatively hydrophilic nature and good water adsorption properties of corn starch films. The outer alginate layer in the HPMC/SA films exhibited a relatively small decline in contact angle from 52.50° to 41.30°, suggesting that it had improved water resistance compared to the other films.

The wettability values of the composite films depended on the blends of polymers they contained, whereas those of the bilayer films were mainly determined by the wettability of the outer polymer layer [56]. All of the films were predominantly hydrophilic (WCA < 90°), but there were distinct differences in the magnitudes of their WCA values and in the changes in these values over time.

#### 3.2.7. Water Dissolution Properties

The water dissolution properties of the different films depended on their composition, structure, and temperature (Table 3). At room temperature, the monolayer films exhibited diverse solubility behaviors depending on polymer type. The SA films completely dissolved in water at around 47 s, reflecting their high sensitivity to water. The CS films only exhibited a low solubility in water (11.7% after 60 s), which suggested that the dense crosslinking between the amylose and amylopectin molecules in the corn starch films made them relatively resistant to water penetration. The PVA films also exhibited relatively low water solubility (5.0%), which can be attributed to the water-resistant nature of their semi-crystalline structure. In contrast, the HPMC films had a moderately good water solubility (59.9%), which was mainly attributed to the relatively strong hydrophilic nature of these polymers [15].

The composite films exhibited water resistance properties that depended on those of the individual polymers that they were composed of. For instance, the CS+PVA films had a solubility of around 4.6%, which was lower than that of the pure CS and pure CVA monolayer films. The CS+HPMC had a solubility of around 30.0%, which was between that of the pure CS and pure HMPC films. The SA+PVA and SA+HPMC films dissolved more readily, with solubilities of around 58.5% and 72.3%, respectively, which suggests that the hydrophilic alginate molecules dominated the overall solubility characteristics of these blended films. However, the solubilities of these two composite films were again between those of the monolayer films of their individual components. Interestingly, the PVA/CS and PVA/SA bilayer films demonstrated enhanced resistance to dissolution, with solubility percentages of around 10.6% and 56.4%, respectively. Conversely, the HPMC/SA bilayer films dissolved completely, with the water-soluble outer SA layer driving their overall dissolution behavior.

In boiling water, the solubility of most of the films increased significantly. The SA and PVA films completely dissolved after around 13.4 and 30.6 s, respectively, which can be attributed to their strong hydrophilicity. The CS films had almost largely dissolved after 60 s incubation in boiling water, with a solubility of around 72.4%. Interestingly, the solubility of the HPMC films was actually much lower after being incubated in boiling water (5.30%) than being incubated in room temperature water (59.9%), which may have been due to an increase in the hydrophobic effect at higher temperatures. This may have led to hydrophobic attraction between the methyl groups on the HPMC molecules, which reduced their water solubility.

The solubility of the composite films during boiling also depended strongly on their composition. The CS+PVA and SA+PVA films fully dissolved in water within 39.9 and 21.4 s, respectively. Thus, the presence of the PVA increased the water solubility of the CS under these conditions. The CS+HPMC bilayer films only exhibited partial water solubility after boiling for 60 s (42.9%), whereas the SA+HPMC films exhibited very little water solubility (2.79%). These results suggest that the presence of the HPMC molecules greatly reduced the solubility of the other components, which can again be attributed to the hydrophobic nature of these molecules at high temperatures.

The solubility of the bilayer films depended strongly on their compositions and structures, and especially on the nature of their outer layers. For instance, the PVA/CS films (87.6%) and the PVA/SA films (100%), exhibited relatively high solubilities, with the PVA/SA films fully dissolving after around 30.7 s. In contrast, the HPMC/CS films exhibited little water solubility after boiling for 60 s (3.46%), whereas the HPMC/SA films only exhibited partial dissolution (55.20%). Again, this effect may be attributed to the presence of HPMC in these films, which becomes relatively hydrophobic under boiling temperatures.

Overall, SA-based films, composites, and bilayer structures displayed rapid dissolution, particularly in boiling water. This characteristic makes them well-suited for applications requiring quick disintegration, such as single-serving food packaging. The balance between water resistance and solubility can be tailored by adjusting the polymer composition, enabling these films to meet diverse functional requirements.

### 3.3. Identification of Optimum Biodegradable Films

Based on the previous studies, the PVA/SA bilayer film was selected as the optimal formulation for creating biodegradable pouches for seasoning oils. This selection was based on a comprehensive evaluation of the physical, barrier, chemical, thermal, and dissolution properties of the various films tested. Although the SA+PVA blend exhibited higher solubility (Table 3), the PVA/SA bilayer film demonstrated superior overall performance, particularly in terms of barrier properties and structural integrity.

The PVA/SA bilayer film showed excellent water resistance at room temperature, while dissolving rapidly and completely in boiling water. These characteristics are essential for protecting the seasoning oil during storage with the noodles and then releasing it when hot water is poured over the noodles. This pouch ensures complete release of the seasoning oil within a few seconds after pouring on the hot water, which would be more desirable for consumer convenience.

Moreover, the PVA-SA bilayer film exhibited strong UV absorbance at 280 nm and a high light transmittance at visual wavelengths (400–800 nm), making it clear to consumers while effectively protecting light-sensitive ingredients from UV degradation. The low oil permeability of this film also prevents leakage of the seasoning during storage, which is critical for maintaining product quality.

Microstructural analysis revealed a compact and well-integrated structure with strong interfacial adhesion between the PVA inner layer and the SA outer layer. FTIR analysis confirmed the good compatibility between the PVA and SA in these films. Thermal analysis using DSC and DTA demonstrated the good heat resistance of these films, which is important for their applications in foods.

In contrast, while the SA+PVA blend showed higher solubility, it had higher WVP and OIP compared to the PVA/SA bilayer film. These properties are less desirable for packaging applications, as they could lead to moisture migration and oil leakage during storage. Additionally, the bilayer structure of PVA/SA films allows for better control over the film’s properties, as each layer can be optimized for specific functions (e.g., PVA for oil resistance and SA for water solubility). This layered approach provides a more balanced performance compared to composite films, where the properties of the individual components may not be fully optimized.

Furthermore, the components used to formulate the PVA/SA bilayer films have regulatory approval. PVA has been given generally recognized as safe (GRAS) status by the U.S. Food and Drug Administration (GRAS Notice No. 767), where it is approved for use in water-soluble edible films [57]. SA is a natural polysaccharide derived from brown algae that has also been classified as GRAS under the Code of Federal Regulations (CFR), with applications in food as a thickening agent, gelling agent, stabilizer, emulsifier, and film-forming material [58]. Its biodegradability and nontoxicity make it widely accepted for use in edible films and coatings [20,59].

In summary, the PVA/SA bilayer films exhibited the optimum physicochemical, functional and regulatory properties. Consequently, they were selected for developing biodegradable pouches that could protect and deliver seasoning oils.

### 3.4. Characterization of Antioxidant-Enriched Film

**Figure 10:**
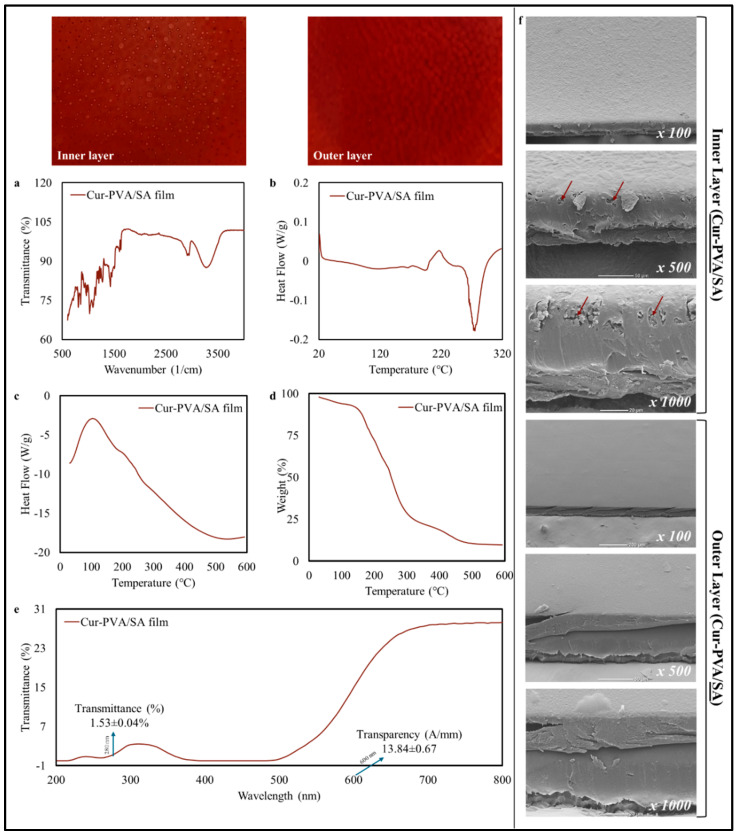
Appearance of both layer, FTIR (**a**), DSC (**b**), TGA (**c**,**d**), light transmittance graphs (**e**) and SEM images (**f**) of PVA/SA film enriched with curcumin (PVA: polyvinyl alcohol, SA: sodium alginate, /: bilayer films as inner/outer).

The possibility of improving the functional performance of the PVA/SA bilayer films by encapsulating a natural antioxidant (curcumin) within them was then examined. It was hypothesized that this natural antioxidant would enhance the quality and shelf life of the seasoning oils. The incorporation of curcumin into the films resulted in significant changes in their physical, chemical, thermal, and barrier properties, as well as their morphology (Figure 10).

#### 3.4.1. Physicochemical Characteristics

*Physical Characteristics:* Cur-PVA/SA films exhibited higher weight (0.679 ± 0.01 g) and greater thickness (0.164 ± 0 mm) than PVA/SA films, which was probably because the presence of the curcumin influenced the nature of the polymer matrix formed in a manner that led to the retention of more material during casting. The curcumin-loaded films were darker and more reddish-yellowish than the control films, as seen by their lower L* (11.47 ± 0.59), higher a* (8.05 ± 0.35), and higher b* (5.09 ± 0.16) values, which can be attributed to the yellow-orange color of curcumin. The relatively large increase in the ΔE* value (85.74 ± 0.58) was consistent with the visible color change in the films containing curcumin, which is consistent with the findings of previous studies [60].

*Dissolution Behavior:* The dissolution time of the Cur-PVA/SA films in hot water was slightly longer (38.7 ± 1.6 s) than that of the PVA/SA films (30.7 ± 2.0 s), which was mainly attributed to the hydrophobicity of curcumin, as well as its ability to alter the interactions between the polymers in the film matrix [61,62]. Despite the longer dissolution time, the curcumin-loaded films still rapidly and fully dissolved in hot water, which is important for their application as pouches designed to release seasoning oils.

*Chemical and Thermal Properties:* FTIR analysis (Figure 10a) confirmed the integration of curcumin into the PVA/SA matrix, with a prominent peak observed at 3300–3400 cm^−1^ (O-H stretching band) and a new peak observed at 1600 cm^−1^ (C=C stretching), which are consistent with the presence of curcumin within the films. A shift in the C=O band at 1730 cm^−1^ suggested hydrogen bonding interactions between the curcumin and polymer network [63]. The DSC thermograms (Figure 10b) suggested that curcumin reduced the glass transition temperature of the films, which may have been a result of its plasticizing effects. The TGA results (Figure 10c,d) showed that the presence of curcumin enhanced the thermal stability of the films, delaying polymer degradation, which may have been due to curcumin’s antioxidant properties, as well as its ability to alter the interactions between the polymers in the film network [64].

*Barrier Properties:* The Cur-PVA/SA films had a lower OIP (0.18 × 10^−6^ g·mm/mm^2^·day), WVP (0.28 × 10^−10^ g/m·Pa·s) and light trasmittance profile (Figure 10e) compared to the control films. This effect may again be attributed to the hydrophobic nature of curcumin, as well as its ability to form hydrogen bonds with the polymers in the film network, thereby creating a more tortuous diffusion pathway for the oil or water molecules to travel through [62,63]. These results suggest that the incorporation of curcumin into the PVA/SA films improved their moisture and oil barrier properties, which would be advantageous for the application of these films for creating pouches to contain seasoning oils. Similarly, Rachtanapun et al. [65] highlighted the potential of natural ingredients with antioxidant properties, such as curcumin, to protect the product, particularly from UV radiation, by reducing light transmittance in films. In addition, the authors reported that curcumin’s improvement in the UV/visible barrier property is related to its phenolic content and the presence of unsaturated bonds in its structure, which is responsible for the absorption of UV/visible radiation.

*Morphological Features:* SEM analysis (Figure 10f) showed that incorporation of curcumin into the bilayer films disrupted the uniformity of the PVA matrix, resulting in rougher surfaces with visible granules, which are consistent with the observations of Xie et al. [63]. However, the outer SA layer remained relatively smooth, with minor irregularities, indicating minimal impact on its morphology. These results suggest that the curcumin may have had stronger interactions with the PVA matrix than the SA matrix.

Many researchers have emphasized that film properties are improved by adding natural bioactive components to the film matrix, and curcumin has been investigated by blending with polymers such as chitosan, pectin, and gelatin [63,65,66,67]. In summary, the presence of curcumin enhanced some of the most important functional attributes of the PVA/SA films, including their barrier and thermal stability properties, without greatly affecting their dissolution behavior. These findings align with previous studies on multi-component hybrid films, which demonstrated superior performance in food packaging applications [63,68,69,70].

#### 3.4.2. Mathematical Diffusion Analysis

**Figure 11:**
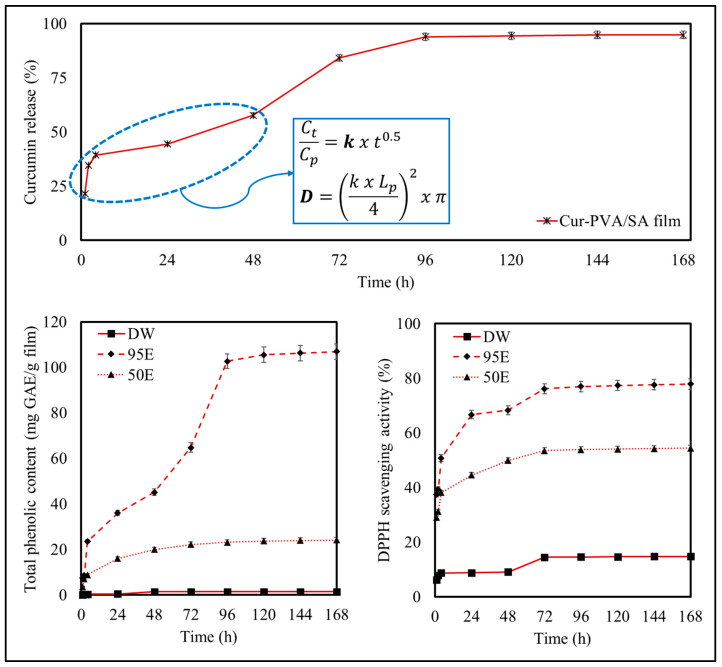
Diffusion kinetics of curcumin from Cur-PVA/SA film and release of TPC and AOA into different food simulants (PVA: polyvinyl alcohol, SA: sodium alginate, /: bilayer films as inner/outer, DW: distilled water as aqueous food, 95E: 95% ethanol as fatty foods, 50E: 50% ethanol as oil-in-water emulsions).

Figure 11 shows the release profile of curcumin from the bilayer films.

Curcumin release exhibited a biphasic release pattern, which was characterized by an initial rapid burst phase followed by a slower sustained release phase. After the first hour, 21.5% of curcumin was released from the films into the release medium, which was mainly attributed to rapid diffusion of curcumin initially located near the surfaces of the films. After 24 h, 44.5% of curcumin was released, and equilibrium was approached at around 72 h. A final cumulative release of 94.8% was observed at 96 h, which remained relatively constant thereafter. The slower release of curcumin observed at later times was attributed to curcumin originally located within the interior of the films. This sustained release behavior may be useful for some applications.

The curcumin release behavior from the films is consistent with the diffusion-controlled release models described by Fick’s laws, where the concentration gradient and diffusivity of the active compound within the polymer matrix govern the release rate. Key factors such as film thickness, porosity, and polymer network density significantly influence the release kinetics. Previous studies have also reported the release kinetics of curcumin from various polymer matrices into different media [8,61,71,72,73].

To further investigate the release mechanism, the effective diffusion coefficient (*D*) of curcumin in the film matrix was estimated as 1.30 × 10^−11^ m^2^/s, using Fick’s second law under transient state conditions. This approach is applicable to the early phases of release, prior to reaching equilibrium, as diffusion continues with different mechanisms when C_t_/C_p_ > 0.6. At this stage, the equation no longer fits the system’s behavior. The calculated *D* value reflects the rate at which curcumin molecules migrate through the film matrix, offering insights into the release mechanism. The relatively low diffusion coefficient suggests that curcumin release is predominantly diffusion-controlled, with the polymeric matrix acting as a barrier to molecular movement [74,75]. This behavior is typical of systems where the active compound is uniformly dispersed within the matrix, and diffusion occurs along the concentration gradient. Additionally, the low *D* value highlights the strong interactions between curcumin and the polymeric components within the film, potentially due to hydrogen bonding and/or hydrophobic interactions. These interactions would reduce curcumin’s mobility, thereby prolonging its release time. Such properties are advantageous for applications requiring sustained curcumin delivery, such as food packaging or nutraceutical films, where prolonged antioxidant activity enhances product stability and shelf life. Xie et al. [63] and Thorat and Dalvi [76] explained this phenomenon as the presence of excess phenolic hydroxyls in curcumin, which can effectively provide hydrogen donors to free radicals and, thus, block the chain reaction.

#### 3.4.3. Release Study to Different Food Simulants

Figure 11 shows the change in the TPC and AOA of substances released from the film matrix into three different food simulants, representing aqueous foods (DW), fatty foods (95E), and oil-in-water emulsions (50E). The release of phenolic compounds from the films displayed solvent-dependent behavior, with significantly higher release observed in 95E compared than the other simulants. This increased release can be attributed to the strongly hydrophobic nature of curcumin and other phenolic compounds, which exhibit higher solubility in ethanol than in water due to their non-polar characteristics. Moreover, ethanol disrupts the interactions between the phenolic compounds and the film matrix, thereby facilitating their diffusion into the solvent. Intermediate TPC release values were observed for the 50E simulant, because the mixed ethanol-water solvent provided moderate solubility to the phenolic compounds while still maintaining some interactions with the film matrix, resulting in a more controlled release profile. The lower release of TPC in the DW simulant can be attributed to the limited solubility of hydrophobic curcumin and other phenolic compounds in aqueous environments, which restricts their diffusion from the film matrix.

These differences in TPC release indicate that solvent polarity plays a crucial role in the release of phenolic substances. Ethanol (95E) weakens the attractive interactions between the phenolic substances and the polymer network, as well as increasing their solubility, thereby promoting their release from the films. In contrast, water (DW) strengthens the hydrophobic attraction between the phenolic substances and the polymer network, as well as decreasing their solubility, thereby reducing their release from the films.

The antioxidant activity of the substances released from the films followed a similar trend to the TPC (95E > 50E > DW), highlighting the relationship between phenolic compound concentration and antioxidant activity. In other words, the greater the amounts of phenolic substances released from the films, the higher the antioxidant activity. Other researchers have reported similar behavior for curcumin encapsulated in other kinds of delivery systems [26,72].

### 3.5. Solubility Behavior of Pouches Containing Single-Serving Seasoning Oil

**Figure 12:**
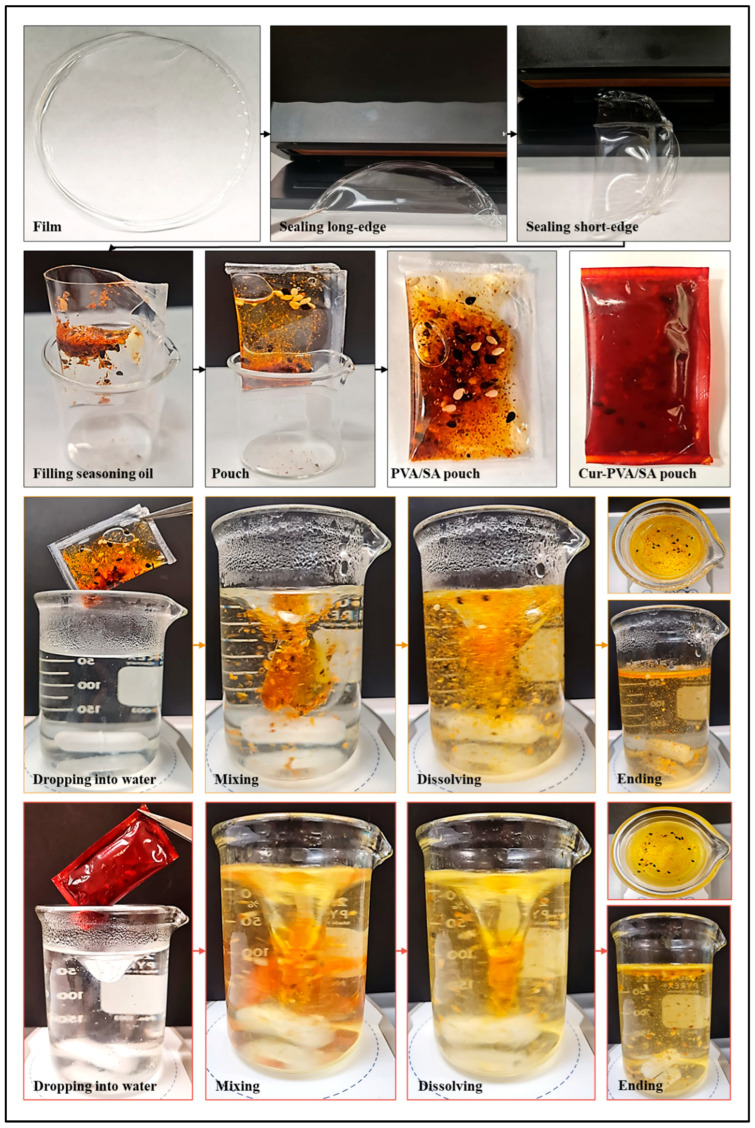
Preparation of pouch containing single-serving seasoning oil from PVA/SA film and its solubility behavior in hot water (PVA: polyvinyl alcohol, SA: sodium alginate, /: bilayer films as inner/outer, Cur: curcumin).

**Figure 13:**
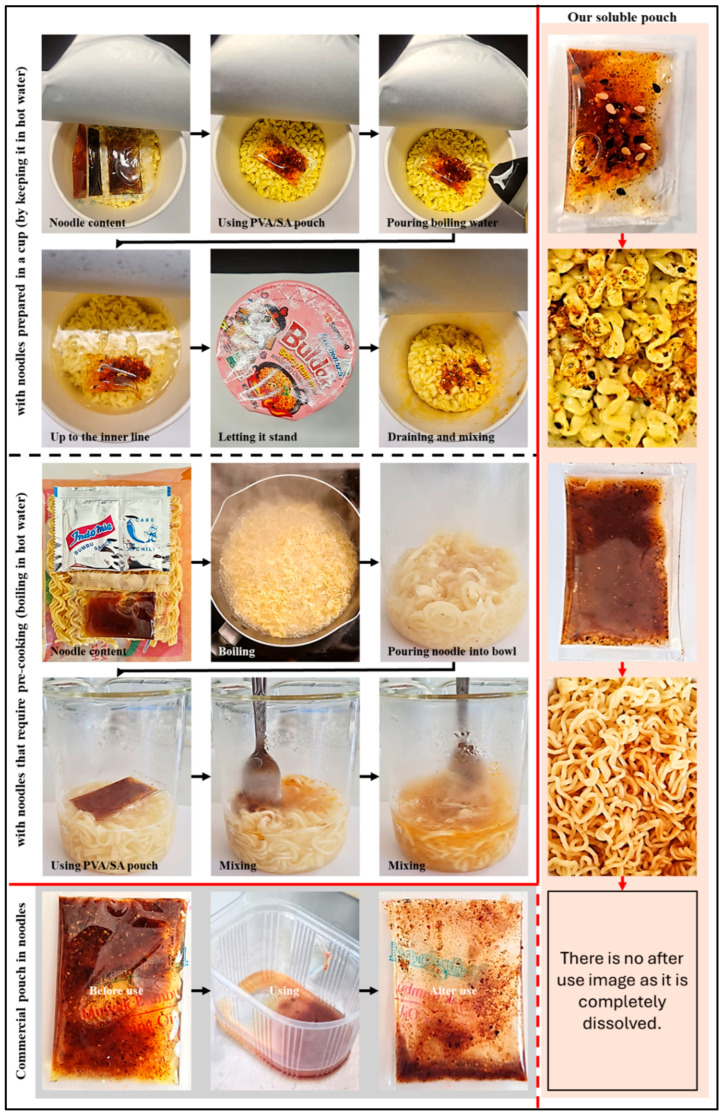
Testing of PVA/SA pouches containing single-serving seasoning oil with 2 different types of noodles (PVA: polyvinyl alcohol, SA: sodium alginate, /: bilayer films as inner/outer).

Pouches were produced from PVA/SA and Cur-PVA/SA films and utilized for single-serving seasoning oil packaging, as shown in Figure 12. These pouches were immersed in hot water, and their dissolution process was monitored. Upon contact with hot water, the PVA/SA pouch began to break down within a few seconds, and the oil mixture started dispersing into the water. The complete dissolution of the PVA/SA pouch occurred within approximately 30 s, as seen in the visual images of these samples. In contrast, the Cur-PVA/SA pouches exhibited slower dissolution behavior. This delay in dissolution is likely due to the effect of curcumin on the polymer matrix, which may have increased the strength of the attractive interactions between the polymer molecules, thereby making the films more resistant to rapid water penetration. As a result, the Cur-PVA/SA pouch disintegrated more gradually, with the oil mixture dispersing more slowly compared to the control PVA/SA pouch. Nevertheless, complete dissolution of the Cur-PVA/SA pouch occurred in approximately 35 s, which aligns with the findings from the Cur-PVA/SA film characterization. The use of these pouches with noodles is also given in Figure 13; the application was very successful.

### 3.6. Storage of Seasoning Oil-Containing Pouches in the Presence of Noodles

For commercial applications, it is important that the seasoning oil-containing pouches remain intact when they are stored in the presence of dried noodles. The change in weight of the seasoning oil-containing pouches and the water activity of the noodles were, therefore, monitored at room temperature to assess moisture migration between the pouches and the noodles.

For the control PVA/SA pouches, only a small weight loss (5.80 ± 0.30%) was recorded after storage for 14 days, which was likely due to moisture migration from the pouches to the noodles. Concurrently, the water activity of the noodles increased from an initial value of 0.40 ± 0.01 to 0.53 ± 0.02, suggesting that the noodles absorbed moisture from the surrounding environment, including from the oil-containing pouches. The presence of curcumin in the PVA/SA pouches enhanced their barrier properties, resulting in a considerably lower weight loss (2.73 ± 0.21%) after 14 days of storage. Indeed, this weight loss was about half that observed for the control pouches. This slower weight loss can be attributed to the hydrophobic nature of curcumin, as well as its ability to promote greater crosslinking between the polymer molecules in the films, thereby reducing the water vapor permeability. Moreover, the water activity of the noodles stored in the presence of Cur-PVA/SA pouches showed a smaller increase, only reaching a water activity of 0.46 ± 0.01 at the end of the storage period.

### 3.7. Quality Changes in Oil Packaged in Pouches During Storage

**Figure 14:**
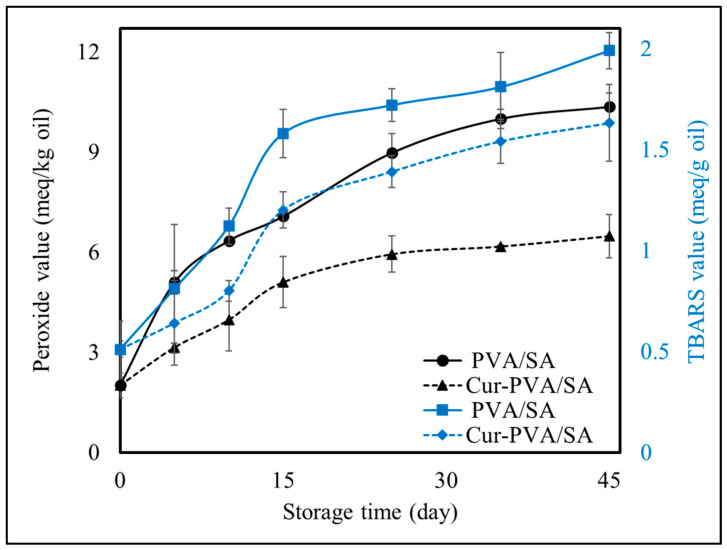
Oxidative stability of sunflower oil in PVA/SA and Cur-PVA/SA pouches during storage at 35 °C (PVA: polyvinyl alcohol, SA: sodium alginate, /: bilayer films as inner/outer, Cur: curcumin).

Finally, the ability of the curcumin-loaded pouches to increase the quality and shelf life of the oil by inhibiting its oxidation was investigated. In these experiments, pure sunflower oil (rather than seasoning oil) was used to avoid the potential impact of the herbs and spices on lipid oxidation. The changes in PV and TBARS during storage for sunflower oil loaded into either PVA/SA or Cur-PVA/SA pouches at 35 ± 2 °C and 50 ± 2% RH are shown in Figure 14. Both pouch types showed an increase in PVs over the storage period, indicating progressive lipid oxidation of the packaged sunflower oil. However, there were differences in the levels of lipid oxidation markers measured for the different pouches.

In the PVA/SA pouches, the PV steadily increased throughout storage, reaching 10.35 meq/kg on day 45. While this value remains below the Codex Alimentarius [77] limit of 20 meq/kg for refined sunflower oil, it indicates substantial oxidation over the storage period. In contrast, the PV for oil stored in the Cur-PVA/SA pouches increased at a significantly slower rate, reaching only 6.48 meq/kg on day 45. The incorporation of curcumin into the bilayer film effectively delayed lipid oxidation by quenching free radicals and stabilizing hydroperoxides, as reported by Dong et al. [8]. Furthermore, curcumin’s UV-blocking capacity may have reduced photo-oxidation, offering additional protection to the oil [11,78].

Our findings are consistent with previous studies that have demonstrated the effectiveness of various edible film pouches in delaying lipid oxidation during oil storage. For instance, Dong et al. [8] reported that soybean oil packaged in soybean polysaccharide and gelatin-based water-soluble pouches exhibited delayed oxidation compared to unpackaged oil, with PVs decreasing from 19.8 meq/kg to 12.8 meq/kg when curcumin was incorporated into the pouches. Similarly, Cho et al. [79] observed PVs ranging from 54 to 93 meq/kg for olive oil stored in corn zein/soy protein isolate pouches after 120 days, demonstrating significant oxidative stability. Rosenbloom and Zhao [11] observed PVs between 33.2 and 47.6 meq/kg for safflower oil stored in hydroxypropyl methylcellulose-based film pouches after 60 days, while Carpiné et al. [80] reported approximately 15% lower PV in soy protein-based film pouches compared to the control for olive oil after 7 days. Hromiš et al. [10] demonstrated that pumpkin oil cake film pouches effectively slowed the formation of conjugated dienes and trienes in flaxseed oil, correlating with lower PVs. Additionally, Nilsuwan et al. [13] found that chicken skin oil stored in gelatin-based mono- and bilayer film pouches showed slower increases in PV and TBARS compared to oils stored in commercial LDPE packaging. The inclusion of epigallocatechin gallate further reduced PV and TBARS values, attributed to its antioxidant release into the oil. Bilayer film pouches were noted to outperform mono-layer films, likely due to enhanced oxygen and water vapor barrier properties.

In our study, the use of PVA/SA bilayer films with curcumin not only provided superior oxidative stability but also demonstrated the potential for long-term storage applications. The Q_10_ model was used to estimate the equivalent storage time of the sunflower oil-loaded pouches under ambient conditions (23 °C). Previously, a Q_10_ value of 2.15 has been reported for sunflower oil [11]. The time required to reach the recommended upper PV limit of 20 mEq/kg was taken to represent the end of the shelf-life of the sunflower oil. The calculations indicated that a storage time of 45 days at 35 °C was equivalent to approximately 7 and 12 months at ambient temperature (23 °C) for PVA/SA and Cur-PVA/SA pouches, respectively. Thus, the incorporation of curcumin into the PVA/SA pouches extended the shelf life of the sunflower oil 1.6-fold under ambient conditions.

TBARs, which measure secondary oxidation products like malondialdehyde (MDA), followed a similar trend to PV, with higher values observed in PVA/SA pouches compared to Cur-PVA/SA pouches. For PVA/SA pouches, the TBARs reached 1.99 mg MDA/kg oil on day 45, reflecting advanced lipid degradation. In contrast, the TBARs only reached 1.63 mg MDA/kg oil for the Cur-PVA/SA pouches after this time, which can be attributed to the antioxidant effects of curcumin.

The ability of curcumin to inhibit lipid oxidation in packaged oils is consistent with the findings of other researchers. For instance, Dong et al. [8] reported that soybean oil packaged in soybean polysaccharide and gelatin-based water-soluble pouches exhibited delayed oxidation compared to unpackaged oil, with PVs decreasing from 19.8 meq/kg to 12.8 meq/kg when curcumin was incorporated into the pouches.

In summary, the results demonstrate that curcumin-enriched bilayer films significantly enhance the oxidative stability of sunflower oil compared to control films. The extended shelf life under ambient conditions, as estimated through the Q_10_ model, underscores the potential of Cur-PVA/SA pouches for long-term storage applications. By combining antioxidant activity with improved barrier properties, curcumin-enriched films offer a sustainable and functional alternative to conventional synthetic packaging, helping to meet consumer and industry demand for more eco-friendly packaging solutions.

## 4. Conclusions

Water-soluble biodegradable films for single-serving oil packaging applications were successfully fabricated from food-grade polymers. Films with different compositions and structures were initially tested. Among these, bilayer films consisting of polyvinyl alcohol (PVA) as inner layers and sodium alginate (SA) as outer layers (PVA/SA films) demonstrated the best physical, thermal, and barrier properties. These films completely dissolved in hot water within about 30 s, making them suitable for instant noodle applications. These films exhibited low UV transmittance, high optical transparency, and strong resistance to oil penetration (0.18 × 10^−6^ g·mm/mm^2^·day), which are essential for maintaining oil quality during storage.

The addition of curcumin to the PVA/SA matrix (Cur-PVA/SA films) significantly enhanced the functionality of the films. Curcumin incorporation reduced light transmittance, improved thermal stability, and decreased both WVP and OIP. Curcumin release studies showed a biphasic mechanism with an initial burst release followed by a sustained release. The seasoning oil-loaded pouches fabricated from the curcumin-enriched films maintained good structural integrity and resistance to moisture migration during storage with dried noodles. The presence of curcumin in the films also reduced the rate of lipid oxidation in packaged sunflower oil, which was mainly attributed to the inherent antioxidant properties of this natural phenolic compound.

The production of curcumin-enriched PVA/SA biodegradable films is scalable and compatible with industrial film-forming technologies like casting and heat-sealing. Food-grade polymers (PVA, SA, CS, HPMC) and natural antioxidants (curcumin) ensure safety and environmental sustainability. However, challenges such as cost-effective curcumin incorporation, film casting efficiency, and sensory attributes (e.g., taste, odor) must be optimized for consumer acceptance and commercial viability. While promising for single-serving oil packaging, limitations include the need to evaluate long-term stability under real-world storage conditions, optimize mechanical properties (tensile strength, elongation at break) for durability, and assess biodegradability under diverse conditions to confirm sustainability.

Future research should focus on scaling production, evaluating sensory properties, and testing real-world performance. Exploring alternative natural antioxidants or bioactive compounds could further enhance functionality. Addressing these limitations will advance this technology to meet the growing demand for sustainable food packaging.

In conclusion, the biodegradable pouches developed in this study present a sustainable alternative to traditional plastic sachets for oil packaging. By reducing plastic waste and pollution, they address critical environmental concerns while enhancing food safety by eliminating exposure to harmful plastic additives. Additionally, their design improves consumer convenience, particularly for seasoning noodle products. This innovation holds significant potential to advance sustainability and efficiency in the food industry.

## Figures and Tables

**Table 1:** Thickness and weight of films.

Films ^1^*	Thickness (mm)			Weight (g)		
	10 mL	15 mL	20 mL	10 mL	15 mL	20 mL
CS	0.031 ± 0.001 ^d^	0.055 ± 0.005 ^e^	0.077 ± 0.006 ^fg^	0.289 ± 0.001 ^g^	0.335 ± 0.005 ^j^	0.483 ± 0.004 ^e^
SA	0.030 ± 0.004 ^d^	0.043 ± 0.004 ^ef^	0.060 ± 0.002 ^g^	0.236 ± 0.002 ^h^	0.328 ± 0.003 ^j^	0.485 ± 0.013 ^e^
PVA	0.064 ± 0.002 ^b^	0.105 ± 0.007 ^c^	0.206 ± 0.010 ^b^	0.631 ± 0.006 ^a^	0.995 ± 0.001 ^a^	1.329 ± 0.008 ^b^
HPMC	0.025 ± 0.004 ^d^	0.039 ± 0.003 ^g^	0.068 ± 0.006 ^g^	0.278 ± 0.001 ^g^	0.363 ± 0.003 ^i^	0.531 ± 0.006 ^e^
CS+PVA	0.075 ± 0.004 ^ab^	0.132 ± 0.007 ^b^	0.179 ± 0.004 ^c^	0.624 ± 0.005 ^a^	0.879 ± 0.005 ^b^	1.467 ± 0.261 ^a^
CS+HPMC	0.047 ± 0.007 ^c^	0.080 ± 0.005 ^d^	0.113 ± 0.014 ^d^	0.336 ± 0.003 ^e^	0.494 ± 0.001 ^f^	0.673 ± 0.005 ^d^
SA+PVA	0.071 ± 0.008 ^ab^	0.093 ± 0.004 ^cd^	0.110 ± 0.007 ^de^	0.517 ± 0.008 ^b^	0.684 ± 0.009 ^d^	1.212 ± 0.017 ^b^
SA+HPMC	0.026 ± 0.004 ^d^	0.034 ± 0.003 ^fg^	0.068 ± 0.004 ^g^	0.322 ± 0.003 ^f^	0.477 ± 0.004 ^g^	0.621 ± 0.001 ^de^
PVA/CS	0.072 ± 0.003 ^ab^	0.182 ± 0.029 ^a^	0.264 ± 0.025 ^a^	0.467 ± 0.003 ^c^	0.702 ± 0.006 ^c^	0.907 ± 0.008 ^c^
PVA/SA	0.084 ± 0.007 ^a^	0.104 ± 0.004 ^c^	0.186 ± 0.014 ^c^	0.425 ± 0.004 ^d^	0.587 ± 0.010 ^e^	0.813 ± 0.005 ^c^
HPMC/CS	0.034 ± 0.003 ^d^	0.052 ± 0.004 ^ef^	0.088 ± 0.002 ^ef^	0.225 ± 0.008 ^i^	0.336 ± 0.004 ^j^	0.490 ± 0.003 ^e^
HPMC/SA	0.029 ± 0.002 ^d^	0.040 ± 0.003 ^efg^	0.055 ± 0.002 ^g^	0.284 ± 0.008 ^g^	0.405 ± 0.005 ^h^	0.550 ± 0.001 ^de^

^1^ CS: corn starch, SA: sodium alginate, PVA: polyvinyl alcohol, HPMC: hydroxypropyl methylcellulose, “+”: composite films (mixture), “/”: bilayer films (inner/outer). * Composite films involve mixing equal volumes of both polymer solutions. Bilayer films involve casting an outer layer film onto an inner layer film. Different letters in the same column indicate significant variances between samples according to the Duncan test (*p* < 0.05).

**Table 2:** Color characteristics of films.

Films ^1^*	L*	a*	b*	ΔE*	YI	WI
CS	31.96 ± 0.74 ^c^	0.39 ± 0.01 ^b^	−0.33 ± 0.03 ^bc^	64.40 ± 0.72 ^j^	−1.49 ± 0.17 ^b^	31.96 ± 0.74 ^c^
SA	4.75 ± 0.03 ^l^	0.23 ± 0.04 ^d^	−0.53 ± 0.03 ^e^	91.22 ± 0.03 ^a^	−15.84 ± 0.86 ^g^	4.75 ± 0.03 ^l^
PVA	11.54 ± 0.04 ^j^	0.35 ± 0.01 ^c^	−0.83 ± 0.03 ^f^	84.45 ± 0.03 ^c^	−10.23 ± 0.34 ^f^	11.54 ± 0.04 ^j^
HPMC	37.09 ± 0.03 ^a^	0.64 ± 0.02 ^a^	−0.44 ± 0.02 ^d^	59.36 ± 0.03 ^l^	−1.71 ± 0.08 ^b^	37.08 ± 0.03 ^a^
CS+PVA	14.85 ± 0.14 ^g^	0.12 ± 0.02 ^e^	−0.52 ± 0.02 ^e^	81.23 ± 0.13 ^f^	−4.97 ± 0.23 ^d^	14.85 ± 0.14 ^g^
CS+HPMC	15.83 ± 0.04 ^f^	0.22 ± 0.02 ^d^	−0.45 ± 0.02 ^d^	80.27 ± 0.03 ^g^	−4.06 ± 0.19 ^c^	15.83 ± 0.04 ^f^
SA+PVA	10.09 ± 0.12 ^k^	0.36 ± 0.03 ^bc^	−1.07 ± 0.07 ^h^	85.85 ± 0.13 ^b^	−15.19 ± 0.96 ^g^	10.08 ± 0.12 ^k^
SA+HPMC	14.31 ± 0.04 ^h^	0.37 ± 0.01 ^bc^	0.07 ± 0.01 ^a^	81.82 ± 0.03 ^e^	0.73 ± 0.11 ^a^	14.34 ± 0.04 ^h^
PVA/CS	22.05 ± 0.02 ^e^	0.07 ± 0.01 ^f^	−0.32 ± 0.03 ^b^	74.16 ± 0.02 ^h^	−2.05 ± 0.19 ^b^	22.05 ± 0.02 ^e^
PVA/SA	34.58 ± 0.33 ^b^	0.11 ± 0.02 ^e^	−0.38 ± 0.01 ^c^	61.84 ± 0.32 ^k^	−1.56 ± 0.01 ^b^	34.58 ± 0.33 ^b^
HPMC/CS	24.05 ± 0.07 ^d^	0.25 ± 0.01 ^d^	−0.97 ± 0.02 ^g^	72.07 ± 0.07 ^i^	−5.76 ± 0.10 ^e^	24.05 ± 0.07 ^d^
HPMC/SA	13.51 ± 0.45 ^i^	0.12 ± 0.01 ^e^	−0.35 ± 0.01 ^bc^	82.58 ± 0.44 ^d^	−3.70 ± 0.17 ^c^	13.51 ± 0.45 ^i^

^1^ CS: corn starch, SA: sodium alginate, PVA: polyvinyl alcohol, HPMC: hydroxypropyl methylcellulose, “+”: composite films (mixture), “/”: bilayer films (inner/outer). * Composite films involve mixing equal volumes of both polymer solutions. Bilayer films involve casting an outer layer film onto an inner layer film. Different letters in the same column indicate significant variances between samples according to the Duncan test (*p* < 0.05).

**Table 3:** Dissolution behavior of films at room temperature and in boiling water.

Films ^1^*	Test at 25 ± 2 °C		Test at 87 ± 2 °C	
	Dissolution Time (s)	Solubility (%)	Dissolution Time (s)	Solubility (%)
CS	>60	11.73 ± 2.22 ^f^	>60	72.44 ± 3.33 ^c^
SA	47.28 ± 1.97 ^b^	100.00 ± 0 ^a^	13.42 ± 1.83 ^d^	100.00 ± 0 ^a^
PVA	>60	5.03 ± 0.76 ^g^	30.57 ± 5.25 ^b^	97.98 ± 0.56 ^a^
HPMC	>60	59.89 ± 2.10 ^c^	>60	5.30 ± 0.70 ^f^
CS+PVA	>60	4.62 ± 1.41 ^g^	39.85 ± 1.85 ^a^	99.54 ± 0.38 ^a^
CS+HPMC	>60	29.97 ± 3.20 ^d^	>60	42.94 ± 1.71 ^e^
SA+PVA	>60	58.48 ± 2.69 ^c^	21.44 ± 1.54 ^c^	100.00 ± 0 ^a^
SA+HPMC	>60	72.26 ± 3.96 ^b^	>60	2.79 ± 0.17 ^f^
PVA/CS	>60	10.63 ± 1.28 ^f^	>60	87.59 ± 2.33 ^b^
PVA/SA	>60	56.36 ± 5.60 ^c^	30.67 ± 2.00 ^b^	99.45 ± 0.51 ^a^
HPMC/CS	>60	22.48 ± 3.15 ^e^	>60	3.46 ± 0.48 ^f^
HPMC/SA	52.45 ± 1.99 ^a^	100.00 ± 0 ^a^	>60	55.20 ± 4.48 ^d^

^1^ CS: corn starch, SA: sodium alginate, PVA: polyvinyl alcohol, HPMC: hydroxypropyl methylcellulose, “+”: composite films (mixture), “/”: bilayer films (inner/outer). * Composite films involve mixing equal volumes of both polymer solutions. Bilayer films involve casting an outer layer film onto an inner layer film. Different letters in the same column indicate significant variances between samples according to the Duncan test (*p* < 0.05).

## Data Availability

Data will be made available on request.
